# Mapping and population size estimates of people who inject drugs in Afghanistan in 2019: Synthesis of multiple methods

**DOI:** 10.1371/journal.pone.0262405

**Published:** 2022-01-28

**Authors:** Abdul Rasheed, Hamid Sharifi, Paul Wesson, Sayed Jalal Pashtoon, Fatemeh Tavakoli, Nima Ghalekhani, Ali Akbar Haghdoost, Alim Atarud, Mohammad Reza Banehsi, Naqibullah Hamdard, Said Iftekhar Sadaat, Willi McFarland, Ali Mirzazadeh

**Affiliations:** 1 Youth Health and Development Organization, Kabul, Afghanistan; 2 HIV/STI Surveillance Research Center, and WHO Collaborating Center for HIV Surveillance, Institute for Futures Studies in Health, Kerman University of Medical Sciences, Kerman, Iran; 3 Department of Epidemiology and Biostatistics, University of California San Francisco, San Francisco, California, United States of America; 4 United Nations Development Programme, Kabul, Afghanistan; 5 Modeling in Health Research Center, Institute for Futures Studies in Health, Kerman University of Medical Sciences, Kerman, Iran; 6 Ministry of Public Health, Kabul, Afghanistan; FHI360, UNITED STATES

## Abstract

**Introduction:**

Mapping and population size estimates of people who inject drugs (PWID) provide information needed for monitoring coverage of programs and planning interventions. The objectives of this study were to provide the locations and numbers of PWID in eight cities in Afghanistan and extrapolate estimates for the country as a whole.

**Methods:**

Multiple population size estimation methods were used, including key informant interviews for mapping and enumeration with reverse tracking, unique object and service multipliers, capture-recapture, and wisdom of the crowds. The results of the several methods were synthesized using the Anchored Multiplier–a Bayesian approach to produce point estimates and 95% credible intervals (CI). Using the prevalence of PWID in the eight cities and their correlation with proxy indicators, we extrapolated the PWID population size for all of Afghanistan.

**Results:**

Key informants and field mapping identified 374 hotspots across the eight cities from December 29, 2018 to March 20, 2019. Synthesizing results of the multiple methods, the number of male PWID in the eight study cities was estimated to be 11,506 (95% CI 8,449–15,093), corresponding to 0.69% (95% CI 0.50–0.90) of the adult male population age 15–64 years. The total number of women who injected drugs was estimated at 484 (95% CI 356–633), corresponding to 0.03% (95% CI 0.02–0.04) of the adult female population. Extrapolating by proxy indicators, the total number of PWID in Afghanistan was estimated to be 54,782 (95% CI 40,250–71,837), men and 2,457 (95% CI 1,823–3,210) women. The total number of PWID in Afghanistan was estimated to be 57,207 (95% CI 42,049–75,005), which corresponds to 0.37% (95% CI 0.27–0.48) of the adult population age 15 to 64 years.

**Discussion:**

This study provided estimates for the number of PWID in Afghanistan. These estimates can be used for advocating and planning services for this vulnerable at-risk population.

## Introduction

Afghanistan, with 37 million people [1], has a concentrated HIV epidemic with a high proportion of cases among people who inject drugs (PWID). UNAIDS estimated 11,000 people were living with HIV in Afghanistan in 2019 with a male-to-female ratio of 2.5:1. The main driver of the HIV epidemic is injection drug use with intersecting factors that include multiple and concurrent sexual partnerships, gender inequalities and violence, and stigma and discrimination [2]. The first cross-sectional surveys to measure HIV prevalence among key populations in Afghanistan were conducted in 2009 in Kabul, Herat, and Mazar-i-Sharif, finding 3.2%, 18.2% and 1.0% of PWID were HIV positive, respectively [3]. Surveys conducted in 5 cities in 2012 found an overall HIV prevalence of 4.4% among PWID, ranging from 0.3% in Mazar-i-Sharif to 13.3% in Herat. The frequency of HIV risk behaviors among PWID also varied across cities; PWID survey results in Kabul showed that 88.5% had inadequate knowledge of HIV transmission, 36.0% reported no condom use at last sex, 0.8% ever shared non-sterile injection equipment, 29.3% ever paid for sex, and 9.4% had symptoms of sexually transmitted infections in the past 12 months [4]. Awareness of harm reduction services ranged from 2.6% in Charikar to 84.3% in Mazar-i-Sharif and ever testing for HIV ranged from 6.8% in Kabul to 70.9% in Herat [4].

The number of PWID living in Afghanistan, the country that produces over 80 percent of the world’s opium [5], has been variously estimated. The most recent study estimated between 2.5 and 2.9 million drug users (about 11% of the population) were living in Afghanistan, with the majority (1.9 to 2.3 million) ingesting or inhaling opiates [5]. The surveys of PWID in 2012 included the unique object multiplier method for size estimation and projected 12,541 PWID in Kabul, 1,211 in Herat, 1,496 in Mazar-i-Sharif and 1,471 in Jalalabad [4]. A survey by the United Nations Office on Drugs and Crime (UNODC) estimated there were between 18,000 and 23,000 PWID in Afghanistan in 2009 [6]. A mapping exercise in 2008 located 1,251 PWID in Kabul and 159 in Jalalabad, with an overall extrapolation of 0.22% adult men being PWID in Afghanistan [7].

There remains a strong need for recent, rigorous estimates of the number of PWID in Afghanistan to help guide the national HIV response. Accurate size estimates provide program staff and policymakers information on the scope of the HIV epidemic, which assists them in planning interventions, setting targets, allocating resources, and monitoring coverage of programs. The objective of the current study was to provide the locations and population size of PWID in eight cities in Afghanistan. To improve rigor, we employed several different population size estimation methods and a Bayesian approach to synthesize the results of all the methods. We also use the estimates from the eight cities with proxy indicators available in all cities to extrapolate findings to an overall estimate of PWID in Afghanistan.

## Methods

### Study sites

**Fig 1:**
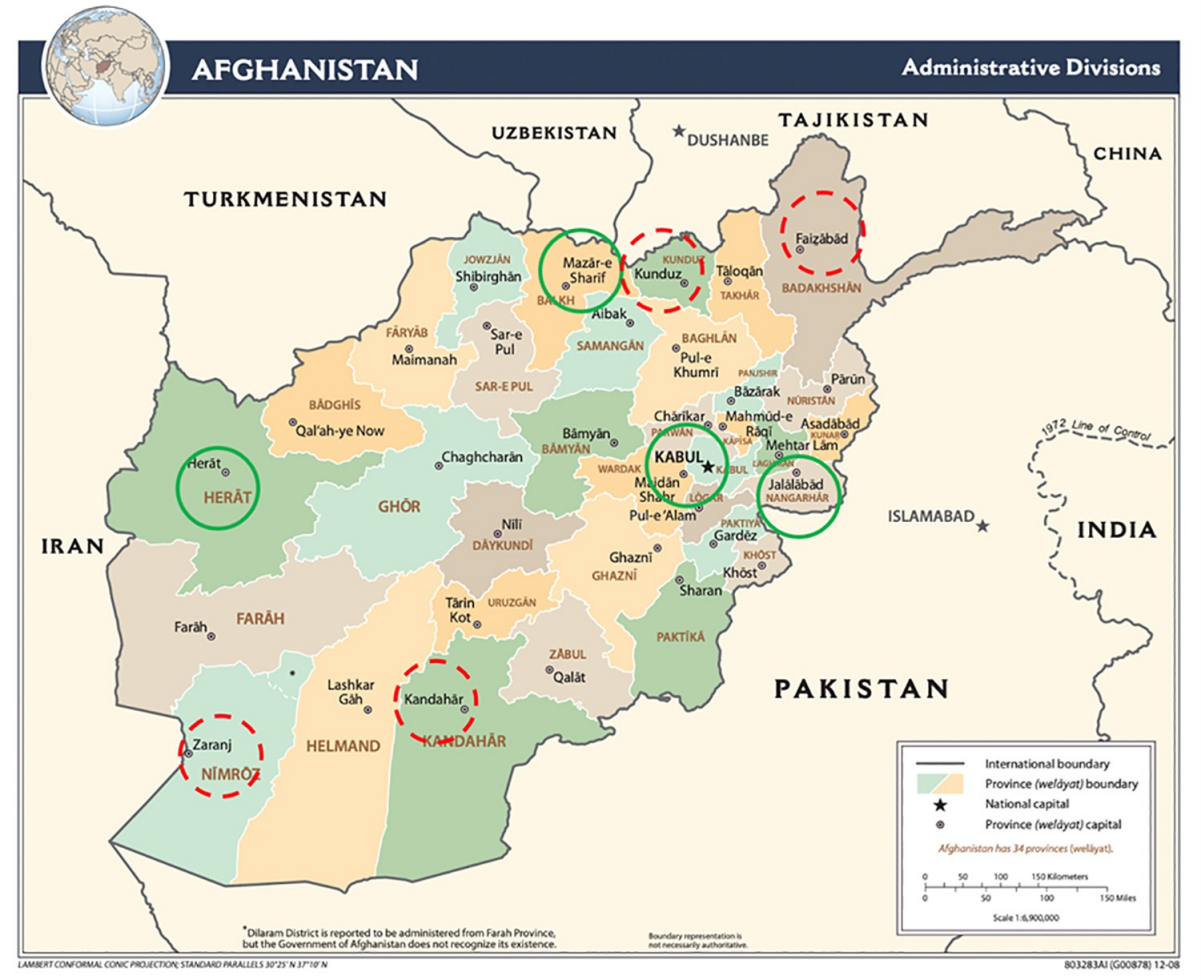
Location of study sites, Afghanistan, 2019 (green circles: A prior population size estimation of people who inject drugs was also conducted in these sites). Republished from [30] under a CC BY license, with permission from Central Intelligence Agency (CIA), original copyright 2009.

Eight cities (Kabul, Herat, Mazar-I-Sharif, Jalalabad, Kunduz, Faizabad, Kandahar, and Zaranj) (Fig 1) were selected to provide a relatively representative cross-section of the regions of Afghanistan. These cities include the major linguistic and geographic zones of the country. City selection also considered available logistical support, safety and security, and the presence of referral services (e.g., harm reduction and other prevention and care services for PWID). In aggregate, these cities comprise 88% of the urban population and 41% of all adults in Afghanistan. These eight cities also include all study sites where previous population size estimation exercises had been conducted.

### Study population

For size estimation, we defined PWID as a person aged 15 to 64 years old who had injected any type of drug at least once for non-medical purposes in the past 12 months. Key informants were included in several methods, including professionals (e.g., health care workers, governmental and non-governmental staff) and PWID community members. PWID community key informants were 18 years of age or older and reported injecting illicit drugs in the past 12 months. PWID key informants also had to not exhibit violent or erratic behavior, and not be so visibly impaired under the influence of drugs to be able to provide informed consent.

### Overview of size estimation methods

We used multiple size estimation methods as recommended by UNAIDS guidelines [8], and applied Bayesian and Delphi methods for triangulation and data synthesis. Methods included: (i) key informant interviews with mapping and enumeration for the reverse tracking method (RTM) [9], (ii) unique object and service multiplier methods [10], (iii) capture-recapture (CRC) using log linear regression models and Bayesian model averaging [11], (iv) wisdom of crowds (WOTC) [12], and (v) a synthesis of the results of all methods using the Anchored Multiplier [11]. The Anchored Multiplier is a Bayesian approach which synthesizes multiple population size estimates coupled to a prior estimate to arrive at a single consensus estimate and a 95% credible interval (CI). Lastly, using the synthesized estimates for PWID in the eight study cities and their correlations with proxy indicators (e.g., population size, literacy, unemployment, etc.), we extrapolated results to produce PWID population size estimates for all Afghanistan. All forms and questionnaires in English, Dari, and Pashtoo are presented in S1-S3 Appendices.

During field observations, we were only able to count PWID present in hotspots at either one or two points in time. We therefore used key informant interview data to correct for under- or over- estimation due to fluctuations in PWID present and to estimate the variance for the number of PWID at each hotspot. Because we found few female PWID present in hotspots (9 total across all 8 cities), direct estimates of the female PWID population size were not possible for most methods. By key informant interviews, focus group discussions, and the wisdom of the crowd method, a male-to-total ratio of PWID in each city was arrived at and used to estimate the number of female PWID.

### Key informant interviews for mapping and enumeration with reverse tracking

The methods of key informant mapping and enumeration with reverse tracking entails compiling information on the locations where PWID congregate, on what days and times they are present, and estimating their numbers at the identified hotspots. Individuals (n = 217) with first-hand knowledge of PWID were interviewed individually or participated in focus group discussions (FGDs) (Table 1). These persons represented non-governmental organizations (NGOs), government officials, health authorities, drop-in center (DIC) staff, or were PWID or former PWID. Key informants were invited from areas throughout the city and asked to identify hotspots where PWID are known to visit, peak hours of activity (i.e., the days of the week and times of day when they are most likely to visit the hotspot), and the approximate number of PWID that can be found at each hotspot. Peers (current or past drug users) and other outreach workers were trained in a 3-day workshop on how to conduct the interviews and complete the data collection forms. Data on demographic information, size estimation, and behaviors related to drug use were collected. Data collection was monitored by a field supervisor on daily basis, and by the team leads assigned to each city on a weekly basis. A technical advisory committee that included HIV surveillance experts from the Ministry of Public Health also visited study sites to ensure completeness and quality of data. Submitted field reports were reviewed at weekly meetings by the principal investigator, country director, and team leads making data corrections and clarifications accordingly. Key informant interviews were conducted from December 29, 2018 to March 20, 2019.

**Table 1:** Number of interviews, hotspots visited, and unique objects distributed to estimate the number of people who inject drugs (PWID) in different cities in Afghanistan, 2019.

	Kabul	Herat	Mazar-I-Sharif	Jalalabad	Kunduz	Faizabad	Kandahar	Zaranj	Total
Number of key informant interviews (individually or in groups) to identify hotspots:									
	30	22	25	30	35	20	25	30	**217**
Number of PWID interviews to define demographic characteristics and population size:									
First survey of PWID:									
	400	200	150	150	151	43	150	150	**1,394**
Second survey of PWID:									
	199	35	80	79	82	23	81	96	**675**
Number of hotspots identified:									
	108	55	41	27	47	17	43	36	**374**
Number of hotspots visited:									
	75	54	41	27	39	17	40	29	**322**
Number of unique objects (winter hats) distributed									
	502	120	150	120	118	35	118	111	**1,274**

The information collected from key informants was summarized in a master list of hotspots that served as a sampling frame for the enumeration phase of data collection. The field team used a mobile-based global positioning system (GPS) app called “AndLocation” to assign coordinates to the location of each hotspot. While visiting hotspots, the field team asked about other locations that were not previously known to the research team and added them to the master list to be visited. For hotspots that were not visited by our team (e.g., due to security issues, or if the hotspot was not selected at random to be visited), their locations or addresses as reported by key informants were geocoded using the offline format of the AndLocation app. The accuracy of the mapped locations was verified by the team lead for each city and research study team. If a named address did not match a geolocation, the city team lead was asked to investigate and assign the correct geocodes. To preserve the confidentiality of data, the maps are not presented in this paper.

Field teams visited all hotspots mentioned by key informants or a random subset of the hotspots in a study site if 50 or more hotspots were mentioned. Field teams visited at least five hotspots in every municipal district at their peak time based on information from key informant interviews and with guidance from the team lead of the city. If more than one peak time was reported for a hotspot, they selected one peak time at random. If no peak time was reported for a hotspot but several dates/times of activity were reported, the field team selected one at random. City team leads developed weekly schedules for hotspot visits using the master list.

The field team, comprised of two data collectors with one acting as field manager, visited each hotspot for a minimum of two hours. At each location, a peer guide (i.e., a current or past drug user) accompanied the team. The field manager counted each person they could identify as a PWID with input from the peer guide. Data collectors interviewed PWID present at the hotspot. The field team, on average, conducted five interviews (range two to eight) with PWID at each hotspot visit, depending on the number present. To improve participation and response rates, we provided cash incentives (around $1 US) to compensate their time for the interview. Because PWID are mobile, it is possible that the enumeration will double count some individuals in more than one hotspot. To try to minimize this error, the survey was implemented in as short a time as possible. The total time in each city was four weeks on average. The numbers of PWID interviewed are reported in Table 1.

**Fig 2:**
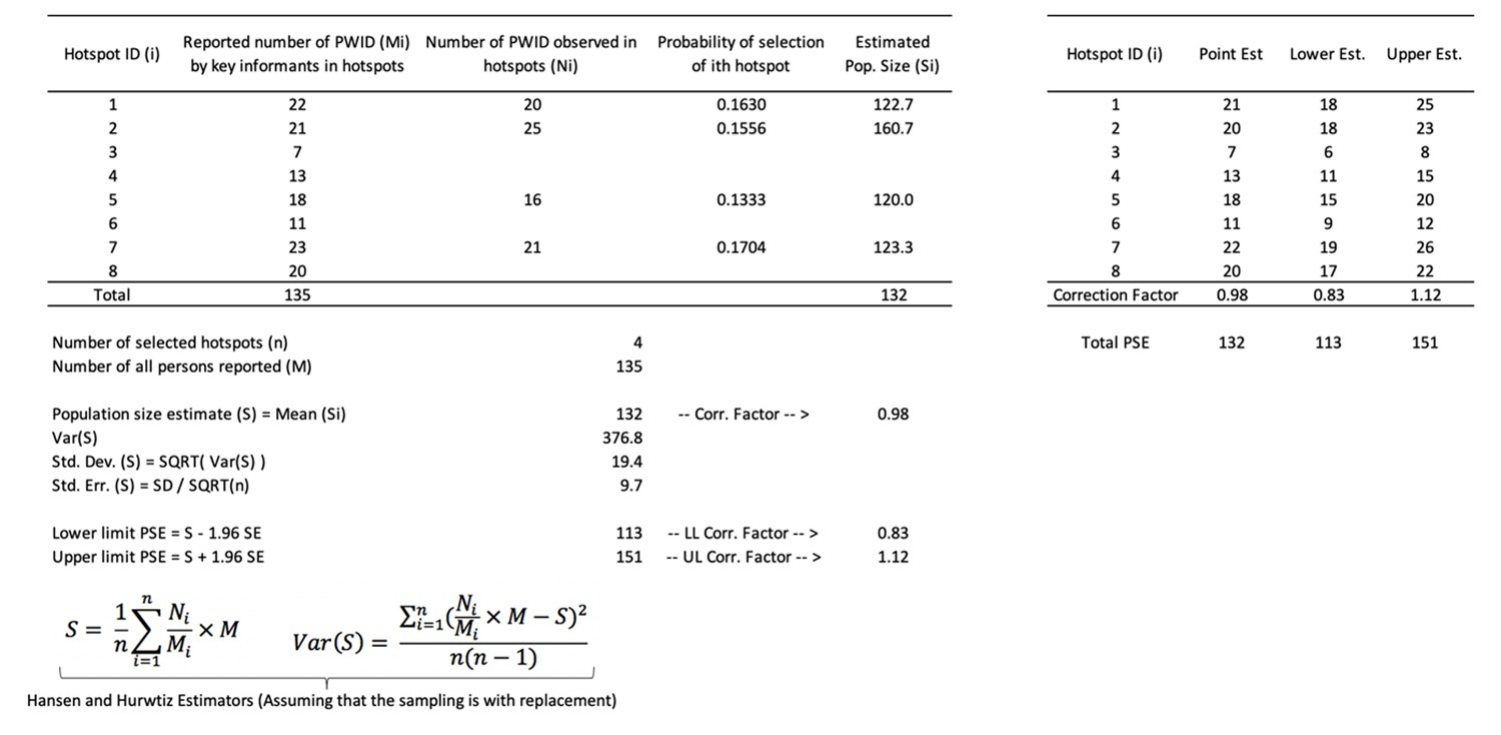
Steps to estimate the number of people who inject drugs in each hotspot by key informant interviews for mapping and enumeration with reverse tracking, Afghanistan, 2019.

To strengthen the population size estimates done by mapping with enumeration, the reverse tracking method (RTM) is used. RTM is done in two stages [9]. In the first stage, key informants provide an approximate count of PWID at each hotspot (Mi, where M is the count of the PWID at each hotspot, indexed by subscript i) (See example, calculations in Fig 2). For the second stage, either all or a random sample of hotspots are visited and the number of PWID present are counted (Ni, where N is the count of the PWID at each hotspot by enumeration, indexed by subscript i). The ratio of Ni/Mi, averaged over all of the hotspots visited, is used as a correction factor that is then multiplied by the sum of the individual counts from the key informant interviews ($M=\sum_{i=1}^{k}M_{i}$) to estimate the total population size. The population size is calculated as follows:

$$S=\frac{1}{n}\overset{n}{\underset{i=1}{\sum }}\frac{N_{i}}{M_{i}}\times M$$

where S is the estimated population size, n refers to the number of hotspots visited, Ni refers to the number of people enumerated at hotspot i, Mi refers to the number of people reported by key informants for hotspot i, and M refers to the total number of people reported by key informants for all hotspots.

The probability of selection of each hotpot is then calculated (e.g., for hotspot 1 = 22 / 135 = 0.1630). The population size (Si) is estimated using data from each hotpot (e.g., for hotspot 1 = 20 / 0.1630 = 122.7). The average of Si is S (= 132) and the variance of Si is Var(S) [= 376.8]. The variance of S is calculated using the following equation:

$$\mathrm{Var}(S)=\frac{\sum_{i=1}^{n}{\left(\frac{N_{i}}{M_{i}}\times M-S\right)}^{2}}{n(n-1)}$$

Using S and its variance, the lower and upper limit of the population size estimate is calculated. These lower and upper limits of the estimated population size (S) were then divided by the total number of persons reported by key informant (M) to calculate the correction factor (Fig 2). This correction factor was then multiplied by each key informant hotspot size to estimate the point estimate for the population size for each hotspot. We used this method to also calculate the correction factor for the lower and upper limits for the size of each hotspot.

### Multiplier methods

As two additional methods to estimate the number of PWID, we used two variations of the multiplier method: the unique object multiplier and the service multiplier. The unique object multiplier entails the distribution of a memorable object (in this case a winter hat) as a “benchmark” count several weeks before the survey data collection at hotspots as described above. The number of distributed unique objects in each city is presented in Table 1. As part of the subsequent field survey, participants were asked: “Did you receive a winter hat, like this hat (show hat), in the past 3 months?” The service multiplier uses a program database from services for PWID to obtain a client count as the benchmark count. Programs in each city were asked to provide unduplicated counts of PWID reached by their program for a clinic visit, HIV testing, or other service in a specified 12-month period. The number of unduplicated counts of PWID reached by programs is presented in S4 Appendix. As part of the field survey, participants were asked: “Did you receive any service from (Center X) in the past 12 months?” If yes, “Which service did you receive from (Center X)? (Select all that apply)”.

To estimate the PWID population size, the multiplier methods use the two data sources, the benchmark count and the survey proportion answering the above questions affirmatively [10]. The benchmark count (n) is the number of PWID who accessed a service (e.g., HIV testing) during the specified timeframe, or the number who received the unique object (the winter hat). The “multiplier” (p) is the proportion of people from the survey of PWID who report receiving the service or receiving the unique object. Dividing the benchmark by the multiplier gives an estimate of the size of the target population (e).


$$Multiplier\;Method=e=\frac{n}{p}$$

Because PWID were recruited from hotspots (i.e., clusters), we used the survey package in R to estimate the lower and upper limits for the 95% confidence interval for p accounting for the clustering effect. We assumed that n is fixed (i.e., has no variation).

### Capture-recapture method

The capture-recapture method uses the overlap of multiple incomplete lists that sample the PWID to estimate the size of the total population. If there is little overlap (i.e., few unique individuals appearing on multiple lists) then the size of the population is estimated to be much larger than what is already observed on the lists. Conversely, if there is a large degree of overlap then the population size is estimated to be not much larger than what has already been observed on the lists. Capture-recapture can be implemented using two lists (analogous to the multiplier method); however, there is greater statistical ability to control for potential biases arising from non-independence of lists when at least three lists are used.

**Fig 3:**
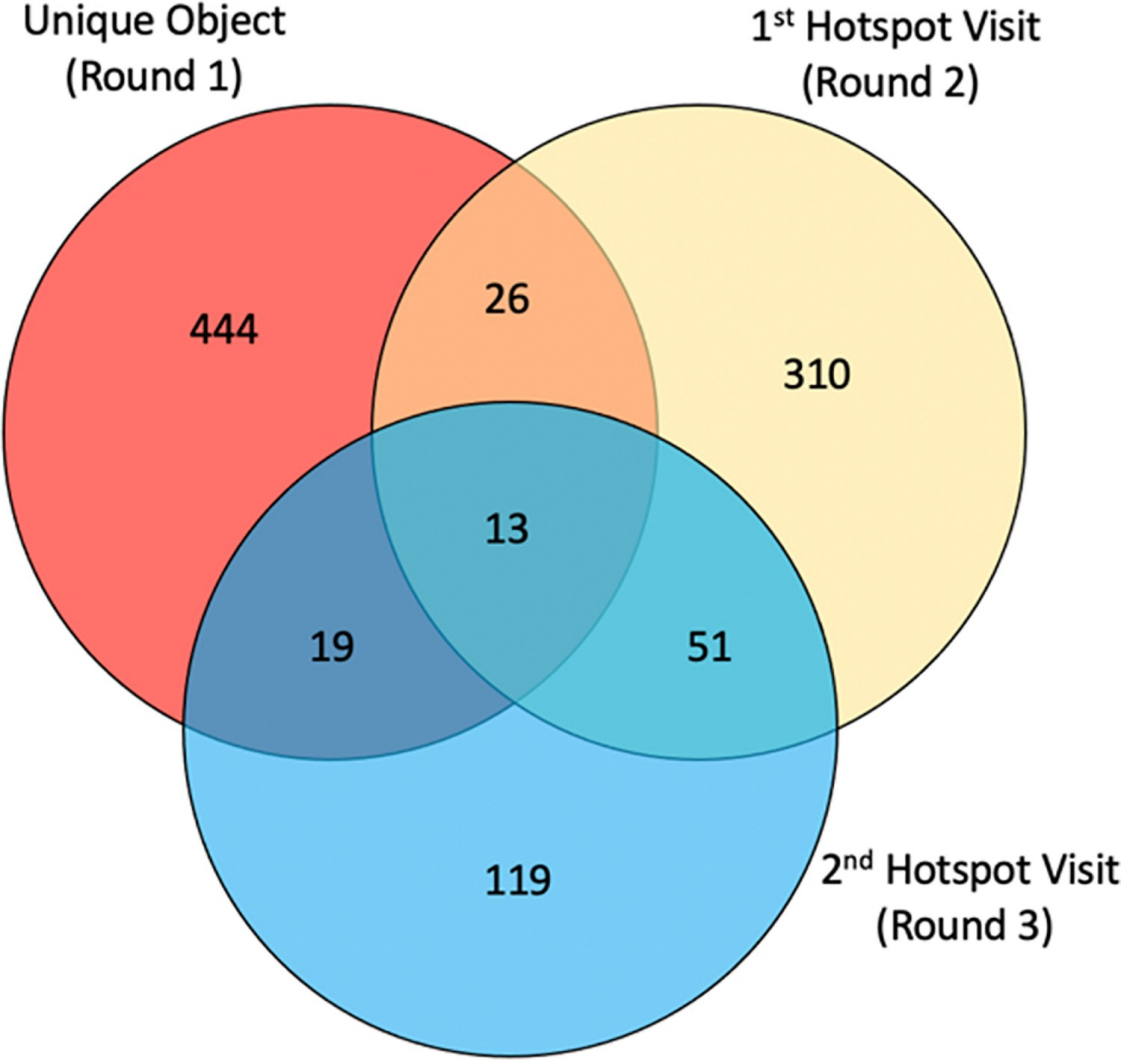
Venn diagram of the 3-round capture-recapture method for estimating the population size of people who inject drugs in Kabul, Afghanistan, 2019.

In this study, we used three capture occasions as the “lists” for the capture-recapture analysis (Fig 3). The first capture occasion came from the distribution of unique objects for the unique object multiplier method. A total of 1,274 winter hats were distributed to PWID in eight cities (Table 1). The second capture occasion came from interviews with PWID at the first visit to hotspots. One of the survey questions from this first visit asked if the respondents received the unique object. If they responded “yes” then they were counted as an overlap between the first capture and the second capture occasions. The third occasion or list was one month after the first visit when the team revisited one third of the hotspots in each district in each city. These hotspots were selected using simple random sampling of one third of the hotspots from the master list. As during the mapping described above, the field team counted each person they could identify as a PWID, and invited them to complete a short survey. In the survey, PWID were asked if they had received the winter hat (assessing the overlap between the first and third capture occasion), and if they had participated in the previous survey administered by the team in the past month (assessing the overlap between the second and third capture occasion). The overlap of all three capture occasions was given by respondents who received the winter hat and had participated in the previous survey.

Log-linear regression models were used to estimate the population size while accounting for potential biases arising from non-independence of lists. A Venn Diagram (Fig 3), representing data from PWID in Kabul, illustrates the overlap between multiple lists. Interaction terms are used in regression models to control for potential statistical dependencies between lists. For a three-source capture-recapture analysis, eight models are possible and estimated (including the model that assumes statistical independence between lists). The R package, Rcapture [13], was used to run the capture-recapture models. By convention, the model with the lowest information criterion (Akaike information criterion: AIC, or Bayesian information criterion: BIC) is selected as the best-fitting model and, by extension, the best estimate for the population size [14, 15]. However, in the present study, we did not select the model with the lowest AIC or BIC as final. Rather, our final capture-recapture estimates were based on the Decomposable Graph Analysis (DGA) model, which is a Bayesian model averaging approach to capture-recapture analysis. In contrast to the log-linear regression modeling approach where a single best-fitting model is selected to estimate the population size, ignoring the remaining models, the DGA model estimates all models (accounting for all combinations of list dependencies) and the posterior probability distribution of each model. The DGA model then creates a weighted average of the population size estimates from all models, weighted by each model’s marginal likelihood, to create a single posterior probability distribution of the estimated population size. From this distribution, a mean and 95% credible interval is taken to represent the population size estimate. Using the DGA approach, information from all models is used to estimate the population size, not selection of a single model.

### Wisdom of the crowds methods

In the survey administered during the mapping and enumeration exercise, respondents were asked to give their best estimate for the minimum and maximum number of PWID in the entire city. We took the average of the reported minimum and maximum to calculate the average population size reported by each participant. The median of the individual responses (i.e., the median minimum, median average, median maximum) was used as the size estimate and range for the number of PWID in the city. This was considered as the “wisdom of the crowds” estimate for the size of the PWID population [12, 16].

### Anchored multiplier method for data synthesis

The Anchored Multiplier method synthesizes multiple estimates of the size of a population into a single estimate [17]. It uses a Bayesian modeling framework to combine empirical estimates (e.g., population size estimates from different multipliers, or combinations of different methods) with a prior belief (e.g., an estimate from a previous study). The calculator will fit the data input to a beta probability distribution that reflects the certainty (i.e., the strength) of the data point [17]. Data points with narrower confidence intervals will have greater influence on the final estimate than data points with wider confidence intervals. When there is additional variance between the estimated population sizes entered that needs to be considered, the calculator will also provide the variance adjusted estimate (“Anchored Multiplier-VA”). The Anchored Multiplier-VA is more conservative and was used in this study. The calculator is available online at https://globalhealthsciences.ucsf.edu/resources/tools. For the prior, we used the population size estimated for PWID in the surveys conducted in Afghanistan in 2012 [4] (Table 2).

**Table 2:** Population sizes estimated for people who inject drugs (PWID) in different cities in Afghanistan in 2012* using the unique object multiplier method. Estimates were used as priors for the Anchored Multiplier Bayesian synthesis of current estimates.

City	Number of PWID			Subgroup	Urban population 15–64 years**	Prevalence (%) of PWID in urban population		
	Point	Lower bound	Upper bound			Point	Lower bound	Upper bound
Kabul	12,546	6,682	27,292	male	1,125,624	1.11	0.59	2.42
Herat	1,211	958	1,582	male	150,110	0.81	0.64	1.05
Mazar	1,495	1,210	1,895	male	125,377	1.19	0.96	1.51
Jalalabad	1,466	1,069	2,101	male	70,099	2.09	1.52	3.0
**Pooled**	**16,719**	**9,919**	**32,870**	**male**	**1,471,210**	1.14	0.67	2.23

* Source: Integrated Behavioral & Biological Surveillance (IBBS) in 2012, Afghanistan

**Source: Afghanistan Central Statistics Organization (CSO) 2018 projection from census 2003–5.

### Adjustment for winter seasonality

While difficult to quantify, cold weather and other conditions such as snow or heavy rains may affect the number of PWID who congregate at hotspots. Some of the hotspots may also be closed sometimes during winter by floods or harsh weather, as we observed with several hotspots in Kabul. These seasonal effects may reduce the overall number of hotspots, but also may increase the number of PWID in hotspots where it is convenient to gather in a warm place during winter. During the warm seasons, PWID are more spread-out through the city neighborhoods, resulting in the number of hotspots being higher, but smaller on average.

We addressed this seasonality effect using two methods. First, instead of the enumerated population size, we estimate the population size for each hotspot using the RTM method which incorporates key informant information on typical attendance. Second, we looked at the seasonality variation in the number of PWID visiting DICs for services in Faizabad and Kunduz, the two cities where the winter season affects are expected to be the harshest (Table 3). On average, in these two cities, the number of PWID who received services per month during the months that population size exercise data were not collected was 10% greater than during the months that data were collected. Assuming the data for monthly variation in 2018 would be similar to the variation in 2019, we increased the total population size for PWID (in all study and extrapolation sites) by 10% (Table 4).

**Table 3:** Number of people who inject drugs (PWID) receiving services over 12 months in 2018 in Faizabad and Kunduz, Afghanistan.

Month	Faizabad	Kunduz	Current Data Collection Period
January	55	475	Yes
February	53	479	Yes
March	55	479	Yes
April	62	487	No
May	55	506	No
June	68	524	No
July	65	447	No
August	71	515	No
September	76	525	No
October	78	531	No
November	80	582	No
December	86	634	No

**Table 4:** Adjustment factor for seasonality (winter) for the population size estimation of people who inject drugs (PWID), Afghanistan, 2019.

Indicator	Faizabad	Kunduz	Total
Total number of PWID for the months that data collected	163	1,433	**1,596**
Total number of PWID for the months data were not collected	641	4,751	**5,392**
Average number of PWID per month for months data were collected	54.3	477.7	**532.0**
Average number of PWID per month for months data were not collected	71.2	527.9	**599.1**
Ratio (%) of average monthly numbers of PWID for months data were not collected to months data were collected	130%	110%	**110%**

### Extrapolation methods

We used Lasso regression with a Poisson family with a log-link function [18] and male adult population as an offset to select the best proxy predictors (among several candidate predictors listed in S5 Appendix) for the extrapolation of the male adult PWID population size of the eight study cities to (unobserved) cities where we did not directly collect data. Lasso regression selected three proxy predictors, “Proportion unemployed”, “Borders with a city/town that produce or traffic drugs”, and “HIV reported cases”. Only the distribution of “Proportion unemployed” was similar between the study cities and unobserved cities (Mean + SD: 21.5+9.8 vs. 22.7+9.7). Therefore, we excluded the other two predictors (i.e., Borders with a city/town that produce or traffic drugs”, and “HIV reported cases”) from the extrapolation model due to lack of overlapping data (i.e., different distribution) between study cities and the other unobserved cities. In summary, using one proxy predictor (Proportion unemployed), we made three Poisson models to extrapolate the point, upper and lower bound estimate of male PWID population size from the eight study cities to other unobserved cities (the models are presented in S5 Appendix). Then, we applied a male-to-total ratio of 96/100 to calculate the total (male + female) adult PWID population size and, then the female adult PWID population size for each unobserved city. To arrive at the national adult population estimate of PWID, we applied the prevalence of total, male and female PWID in the 31 cities (included cities in the extrapolation) to the corresponding national adult population size.

### Ethical considerations

The study proposal was reviewed and approved by the Internal Review Boards (IRBs) of the Afghanistan National Public Health Institute, Ministry of Public Health (#444899, 12/29/2018) and the University of California San Francisco (#234207, 03/08/2019). Participants were briefed about the study aims, processes, and the anonymous nature of the study. Verbal consent was obtained to preserve anonymity.

## Results

We approached 1,394 PWID in hotspots, of whom 1,378 (98.8%) participated in the survey, including 1,369 men and 9 women (Table 5). Demographically, 42.2% were age 25–34 years, 57.0% were younger that 35 years; 40.8% were single; 67.6% spoke Dari. Most (82.5%) reported they last injected within 3 months and 99.3% reported heroin as their most common drug for injection. Self-reported HIV prevalence was 20.7%, ranging from 0% in Zaranj to 63.0% in Kabul. A majority (82.0%) had ever tested for HIV; 70.0% said they knew their HIV status.

**Table 5:** Characteristics of people who inject drugs (PWID) who participated in the survey by city, Afghanistan, 2019.

Characteristics	Overall		Kabul		Herat		Mazar-I-Sharif		Jalalabad		Kunduz		Faizabad		Kandahar		Zaranj	
	N	%	N	%	N	%	N	%	N	%	N	%	N	%	N	%	N	%
**Sex**																		
Male	1,369	99.3	395	100	199	100	146	99.3	147	100	149	100	34	81.0	149	100	150	100
Female	9	0.7	0	0	0	0	1	0.7	0	0	0	0	8	19.0	0	0	0	0
**Age group (years)**																		
18–24	195	14.2	35	8.9	77	38.7	4	2.7	24	16.3	18	12.1	8	19.0	23	15.4	6	4.0
25–34	581	42.2	143	36.2	54	27.1	72	49.0	77	52.4	83	55.7	20	47.6	60	40.3	72	48.0
35–44	409	29.7	146	37.0	42	21.1	43	29.3	39	26.5	32	21.5	11	26.2	40	26.8	56	37.3
45–54	156	11.3	55	13.9	23	11.6	26	17.7	5	3.4	15	10.1	2	4.8	21	14.1	9	6.0
55+	37	2.7	16	4.1	3	1.5	2	1.4	2	1.4	1	0.7	1	2.4	5	3.4	7	4.7
**Language**																		
Pashtoo	430	31.2	75	19.0	1	0.5	10	6.8	145	98.6	37	24.8	0	0	129	86.6	33	22.0
Dari	932	67.6	320	81.0	198	99.5	124	84.4	2	1.4	110	73.8	41	97.6	20	13.4	117	78.0
Uzbek	16	1.2	0	0	0	0	13	8.8	0	0	2	1.3	1	2.4	0	0	0	0
**Last injected**																		
In 1 month	1,005	72.9	392	99.2	42	21.1	141	95.9	130	88.4	142	95.3	32	76.2	34	22.8	92	61.3
In 3 months	132	9.6	1	0.3	48	24.1	2	1.4	13	8.8	6	4.0	5	11.9	33	22.1	24	16.0
In 12 months	241	17.5	2	0.5	109	54.8	4	2.7	4	2.7	1	0.7	5	11.9	82	55.0	34	22.7
**Drugs injected**																		
Heroin	1,369	99.3	390	98.7	199	100	147	100	147	100	147	98.7	42	100	149	100	148	98.7
Cocaine	22	1.6	3	0.8	0	0	0	0	1	0.7	16	10.7	1	2.4	1	0.7	0	0
Opium	54	3.9	6	1.5	0	0	0	0	0	0	25	16.8	4	9.5	18	12.1	1	0.7
Amphetamines	4	0.3	0	0	0	0	0	0	0	0	4	2.7	0	0	0	0	0	0
Prescription	49	3.6	1	0.3	0	0	10	6.8	2	1.4	12	8.1	0	0	24	16.1	0	0
Others	404	29.3	279	70.6	0	0	45	30.6	0	0	43	28.9	1	2.4	0	0	36	24.0
**Marital status**																		
Single	562	40.8	145	36.7	97	48.7	52	35.4	40	27.2	84	56.4	28	66.7	59	39.6	57	38.0
Married living with partner	424	30.8	144	36.5	15	7.5	19	12.9	100	68.0	50	33.6	7	16.7	61	40.9	28	18.7
Married, not living with partner	301	21.8	99	25.1	76	38.2	61	41.5	3	2.0	14	9.4	2	4.8	9	6.0	37	24.7
Not married, living with partner	3	0.2	0	0	0	0	0	0	0	0	0	0	1	2.4	2	1.3	0	0
Separated, divorced	52	3.8	4	1.0	5	2.5	13	8.8	0	0	1	0.7	2	4.8	9	6.0	18	12.0
Widowed	36	2.6	3	0.8	6	3.0	2	1.4	4	2.7	0	0	2	4.8	9	6.0	10	6.7%

Because we found few women PWID at hotspots (9 across all 8 cities), direct estimates of the female PWID population size were not possible for most methods. By key informant interviews and focus group discussions, and using the wisdom of the crowd method, a male-to-total ratio of PWID in each city was used to estimate the number of female PWID (Table 6) and incorporated into the further synthesized and extrapolated estimates.

**Table 6:** Number of men and women who inject drugs and male to total ratio estimated by key informants and wisdom of the crowds methods, by city, Afghanistan, 2019.

City	Key informant interviews, focus group discussions			Wisdom of the crowd’s estimation			Average of the two methods		
	Male	Female	Male/ Total ratio	Male	Female	Male/ Total ratio	Male	Female	Male/ Total ratio
Kabul	1,185	4	100%	4,615	234	95%	2,900	119	96%
Herat	345	9	97%	360	5	99%	353	7	98%
Mazar	420	1	100%	201	7	97%	311	4	99%
Jalalabad	232	1	100%	238	10	96%	235	6	98%
Kunduz	485	1	100%	152	2	99%	319	1	99%
Faizabad	36	6	86%	16	4	80%	26	5	84%
Kandahar	258	1	100%	135	2	99%	197	1	99%
Zaranj	102	2	98%	515	134	79%	309	68	82%
**Total**	**3,063**	**25**	**99%**	**6,232**	**397**	**94%**	**4,648**	**211**	**96%**

### Reverse tracking method

A total of 374 hotspots for PWID were identified in the eight cities, of which 322 (86.1%) were visited by the research team (Table 1). Kabul had the highest number of hotspots (108 with 75 or 69.4% visited) and Faizabad had the lowest number (17 with 100% visited). The median number of PWID per hotspot was 11 (IQR 5–32). By the RTM, the population size of male PWID in Kabul was estimated at 7,542 (95% CI 5,178–10,018), corresponding to 0.67% (95% CI 0.46–0.89) of the adult population age 15–64 years (Table 7). The population size estimates across all study cities using the RTM ranged from 0.16% of the adult male population in Herat to 9.34% in Zaranj.

**Table 7:** Population size estimates (PSE) of male people who inject drugs (PWID) as a synthesis of multiple methods using the anchored multiplier bayesian approach, eight cities in Afghanistan, 2019 (unadjusted for winter seasonality).

City	PWID Prevalence (%)			Number of persons		
PSE Methods	Point	Lower Bound	Upper Bound	Point	Lower Bound	Upper Bound
**Kabul (male pop. 1,125,624)**	** **	** **	** **	** **	** **	** **
Prior [4]	1.11	0.59	2.42	12,494	6,641	27,240
RTM	0.67	0.46	0.89	7,542	5,178	10,018
UOM	0.46	0.33	0.76	5,178	3,715	8,555
CRC (DGA)	0.21	0.11	0.33	2,364	1,238	3,715
SM: Free Needle Syringe (MoPH DIC)	0.21	0.17	0.27	2,364	1,914	3,039
WOTC	0.41	0.36	0.46	4,615	4,052	5,178
Anchored Multiplier	0.38	0.34	0.42	4,277	3,827	4,728
**Anchored Multiplier Variance Adjusted**	**0.47**	**0.33**	**0.64**	**5,290**	**3,715**	**7,204**
**Herat (male pop. 150,110)**						
Prior [4]	0.81	0.64	1.05	1,216	961	1,576
RTM	0.16	0.13	0.19	240	195	285
UOM	0.29	0.23	0.38	435	345	570
CRC (DGA)	0.58	0.28	0.74	871	420	1,111
SM1: Any service (BDN)	0.06	0.06	0.07	90	90	105
SM2: Any service (MoPH DIC)	0.15	0.14	0.16	225	210	240
WOTC	0.24	0.21	0.27	360	315	405
Anchored Multiplier	0.12	0.11	0.12	180	165	180
**Anchored Multiplier Variance Adjusted**	**0.41**	**0.33**	**0.5**	**615**	**495**	**751**
**Mazar (male pop. 125,377)**						
Prior [4]	1.19	0.97	1.51	1,492	1,216	1,893
RTM	0.36	0.30	0.42	451	376	527
UOM	0.25	0.20	0.33	313	251	414
CRC (DGA)	0.65	0.58	0.73	815	727	915
SM2: Free Prevention Education (BDN)	0.54	0.46	0.65	677	577	815
WOTC	0.16	0.14	0.18	201	176	226
Anchored Multiplier	0.32	0.30	0.34	401	376	426
**Anchored Multiplier Variance Adjusted**	**0.83**	**0.65**	**1.04**	**1,041**	**815**	**1,304**
**Jalalabad (male pop. 70,099)**						
Prior [4]	2.09	1.52	3.00	1,465	1,066	2,103
RTM	0.43	0.34	0.52	301	238	365
UOM	0.49	0.36	0.73	343	252	512
CRC (DGA)	1.12	1.02	1.25	785	715	876
SM1: Any services (ADAA)	0.34	0.34	0.36	238	238	252
WOTC	0.34	0.29	0.39	238	203	273
Anchored Multiplier	0.37	0.36	0.38	259	252	266
**Anchored Multiplier Variance Adjusted**	**0.86**	**0.67**	**1.07**	**603**	**470**	**750**
**Kunduz (male pop. 48,871)**						
Prior (expert opinion)	1.75	1.45	2.41	855	709	1,178
RTM	2.12	1.57	2.67	1,036	767	1,305
UOM	0.44	0.37	0.55	215	181	269
CRC (DGA)	1.65	1.56	1.76	806	762	860
SM1: HIV test (YHDO)	3.49	2.51	5.76	1,706	1,227	2,815
WOTC	0.31	0.2	0.41	152	98	200
Anchored Multiplier	1.27	1.19	1.35	621	582	660
**Anchored Multiplier Variance Adjusted**	**1.84**	**1.32**	**2.43**	**899**	**645**	**1,188**
**Faizabad (male pop. 10,189)**						
Prior (expert opinion)	3.83	2.59	4.9	390	264	499
RTM	0.55	0.43	0.67	56	44	68
UOM	0.37	0.34	0.39	38	35	40
CRC (DGA)	2.39	2.22	2.61	244	226	266
SM1:Free Prevention Education (YHDO)	1.2	0.91	1.74	122	93	177
Anchored Multiplier	0.49	0.47	0.52	50	48	53
**Anchored Multiplier Variance Adjusted**	**2.93**	**2.21**	**3.72**	**299**	**225**	**379**
**Kandahar (male pop. 134,936)**						
Prior (expert opinion)	0.72	0.5	1.02	972	675	1,376
RTM	0.19	0.14	0.24	256	189	324
UOM	0.3	0.23	0.41	405	310	553
CRC (DGA)	0.6	0.51	0.78	810	688	1,053
SM1: DIC (ADAA)	1.27	1.08	1.53	1,714	1,457	2,065
WOTC	0.1	0.08	0.13	135	108	175
Anchored Multiplier	0.28	0.25	0.32	378	337	432
**Anchored Multiplier Variance Adjusted**	**0.76**	**0.58**	**0.96**	**1,026**	**783**	**1,295**
**Zaranj (male pop. 8,039)**						
Prior (expert opinion)	6.90	5.06	9.64	555	407	775
RTM	9.34	6.12	12.58	751	492	1011
UOM	3.13	2.46	4.34	252	198	349
CRC (DGA)	9.85	9.17	10.64	792	737	855
SM1: Free Needle Syringe (SHRO)	11.2	10.04	12.65	900	807	1,017
SM2: HCV test (SHRO)	10.46	9.11	12.27	841	732	986
WOTC	6.41	5.6	7.21	515	450	580
Anchored Multiplier	8.74	8.27	9.21	703	665	740
**Anchored Multiplier Variance Adjusted**	**8.53**	**6.6**	**10.57**	**686**	**531**	**850**

Abbreviations: ADAA Agency for Assistance and Development for Afghanistan; BDN Bakhter Development Network; CRC capture-recapture; DGA decomposable graph analysis; MoPH DIC Ministry of Public Health Drop-in Center; PSE population size estimation; PWID people who inject drugs; RTM reverse tracking method; SHRO Shahamat Health and Rehabilitation Organization; SM service multiplier; UOM unique object multiplier; WOTC wisdom of the crowds; YHDO Youth Health and Development Organization.

### Unique object multiplier method

The unique object multiplier method estimated the population size of male PWID in Kabul to be 5,178 (95% CI 3,715–8,555), translating to 0.46% (95% CI 0.33–0.76) of the adult population (Table 7). The range of the prevalence of PWID estimated by the unique object multiplier method ranged from 0.25% in Herat to 3.13% in Zaranj.

### Service multiplier method

In the survey of PWID in Kabul, 38.3% reported use of the free needle and syringe program at the Ministry of Public Health DIC (MoPH DIC) in 2018. The total unduplicated client count was 898 PWID receiving this service. The service multiplier method therefore calculates a PWID population size of 2,364 (95% CI 1,91–3,039) or 0.21% (95% CI 0.17–0.27) of the adult population for Kabul (Table 7). For Herat, service counts from two NGOs calculated male PWID population sizes of 90 (0.06% of adults) using the Bakhter Development Network client count and 225 (0.15% of adults) using the MoPH DIC client count. For other cities, the service multiplier method estimated the prevalence of PWID from 0.34% of adults in Jalalabad to 11.20% in Zaranj.

### Capture-recapture method

The capture-recapture method estimated number of male PWID in Kabul at 2,364 (95% CI 1,238–3,715) persons, corresponding to 0.21% (95% CI 0.11–0.33) of the adult population. The population proportions ranged from 0.58% in Herat to 9.85% in Zaranj.

### Wisdom of the crowds

The median of responses of survey participants for their perception of the number of male PWID in Kabul was 4,615 (95% CI 4,052–5,178), translating to 0.41% (95% CI 0.36–0.46) of the adult population. The prevalence of PWID using the wisdom of the crowds method ranged from a low of 0.10% in Kandahar to a high of 6.41% in Zaranj.

### Anchored Multiplier Variance Adjusted synthesized estimate

Using the Anchored Multiplier Variance Adjusted method to synthesize the results of all the methods, the estimated number of male PWID in Kabul was 5,290 (95% CI 3,715–7,204), corresponding to 0.47% (95% CI 0.33 to 0.64) of the adult population. The prevalence of PWID ranged from a low of 0.41% in Herat to a high of 8.53% in Zaranj.

### Population sizes for the aggregate eight study cities

After adjusting for the winter seasonality effect, the population size of male PWID was 11,506 (95% CI 8,449–15,093) corresponding to 0.69% (95% CI 0.50–0.90) of the adult male population living in these eight cities (Table 8). The total number of women who injected drugs was estimated at 484 (95% CI 356–633), corresponding to 0.03% (95% CI 0.02–0.04) of the adult female population (Table 9). The total population size of PWID was 11,990 (95% CI 8,805–15,726) corresponding to 0.36% (95% CI 0.27–0.48) of the adult population living in these eight cities (Table 10).

**Table 8:** Extrapolated population size and prevalence estimates for male people who inject drugs (PWID) in the adult male population (15–64 years old) in major cities in Afghanistan, and at the national level in 2019 (adjusted for winter seasonality effect).

City	Number of persons			Adult male population	Prevalence (%)		
	Point	Lower Bound	Upper Bound		Point	Lower Bound	Upper Bound
**Extrapolated sites:**							
Asadābād	27	20	36	4,141	0.66%	0.48%	0.87%
Aybak	52	37	69	9,147	0.57%	0.41%	0.76%
Bāmiyān	19	14	26	3,977	0.48%	0.35%	0.66%
Chaghcharān	21	16	27	2,149	0.99%	0.75%	1.26%
Chārīkār	95	69	128	17,584	0.54%	0.39%	0.73%
Farāh	69	51	92	11,150	0.62%	0.45%	0.82%
Gardīz	64	48	83	7,482	0.86%	0.64%	1.10%
Ghaznī	139	103	181	18,228	0.76%	0.56%	0.99%
Khowst	40	31	50	3,504	1.14%	0.87%	1.43%
Lashkar Gāh	226	169	289	24,733	0.91%	0.69%	1.17%
Maḥmūd-e Rāqī	3	2	4	400	0.71%	0.52%	0.93%
Maydān Shahr	8	6	11	955	0.86%	0.64%	1.11%
Mehtarlām	12	9	16	1,514	0.82%	0.61%	1.06%
Meymaneh	210	157	272	25,314	0.83%	0.62%	1.07%
Pol-e ’Alam	14	11	18	1,672	0.85%	0.64%	1.10%
Pol-e Khomrī	128	90	177	32,560	0.39%	0.28%	0.54%
Qalāt	25	19	33	3,425	0.73%	0.54%	0.96%
Qal’eh-ye Now	29	21	38	4,458	0.64%	0.47%	0.85%
Sar-e Pol	43	31	58	9,236	0.46%	0.33%	0.63%
Sharan	9	7	12	1,323	0.69%	0.51%	0.91%
Sheberghān	207	154	267	24,908	0.83%	0.62%	1.07%
Tāloqān	166	122	216	22,437	0.74%	0.55%	0.96%
Tarīn Kowt	20	15	25	2,070	0.96%	0.72%	1.23%
**Subtotal**	1,627	1,200	2,129	232,367	0.70%	0.52%	0.92%
**Study sites:**							
Kabul	5,819	4,087	7,924	1,125,624	0.52%	0.36%	0.70%
Herat	677	545	826	150,110	0.45%	0.36%	0.55%
Mazar	1,145	897	1,434	125,377	0.91%	0.72%	1.14%
Jalalabad	663	517	825	70,099	0.95%	0.74%	1.18%
Kunduz	989	710	1,307	48,871	2.02%	1.45%	2.67%
Faizabad	329	248	417	10,189	3.23%	2.43%	4.09%
Kandahar	1,129	861	1,425	134,936	0.84%	0.64%	1.06%
Zaranj	755	584	935	8,039	9.39%	7.26%	11.63%
**Subtotal**	11,506	8,449	15,093	1,673,245	0.69%	0.50%	0.90%
**In 31 urban sites**	13,133	9,649	17,222	1,905,612	0.69%	0.51%	0.90%
**National (urban and rural)**	54,782	40,250	71,837	7,948,784	0.69%	0.51%	0.90%

**Table 9:** Extrapolated population size and prevalence estimates for female people who inject drugs (PWID) in the adult female population (15–64 years old) in major cities in Afghanistan, and at the national level in 2019 (adjusted for winter seasonality effect).

City	Number of persons			Adult female population	Prevalence (%)		
	Point	Lower Bound	Upper Bound		Point	Lower Bound	Upper Bound
**Extrapolated sites:**							
Asadābād	2	1	2	4,063	0.04%	0.02%	0.05%
Aybak	2	2	4	8,343	0.03%	0.02%	0.05%
Bāmiyān	2	1	2	3,627	0.05%	0.03%	0.05%
Chaghcharān	2	1	2	2,032	0.09%	0.05%	0.10%
Chārīkār	4	3	5	16,396	0.02%	0.02%	0.03%
Farāh	4	2	4	10,882	0.03%	0.02%	0.04%
Gardīz	3	2	3	7,110	0.04%	0.03%	0.05%
Ghaznī	6	5	8	17,339	0.03%	0.03%	0.05%
Khowst	2	1	3	3,410	0.06%	0.04%	0.08%
Lashkar Gāh	10	8	13	21,358	0.05%	0.04%	0.06%
Maḥmūd-e Rāqī	1	1	2	508	0.20%	0.18%	0.39%
Maydān Shahr	1	1	1	871	0.09%	0.10%	0.16%
Mehtarlām	1	1	1	1,524	0.04%	0.05%	0.06%
Meymaneh	10	7	12	24,157	0.04%	0.03%	0.05%
Pol-e ’Alam	1	1	2	1,596	0.05%	0.08%	0.10%
Pol-e Khomrī	6	4	8	31,194	0.02%	0.01%	0.03%
Qalāt	2	1	2	3,281	0.06%	0.04%	0.07%
Qal’eh-ye Now	1	1	2	4,280	0.03%	0.03%	0.05%
Sar-e Pol	2	1	3	8,923	0.02%	0.02%	0.03%
Sharan	1	1	1	1,280	0.06%	0.10%	0.07%
Sheberghān	9	7	12	23,360	0.04%	0.03%	0.05%
Tāloqān	7	6	10	20,821	0.04%	0.03%	0.05%
Tarīn Kowt	1	1	2	1,959	0.06%	0.05%	0.08%
**Subtotal**	78	61	101	218,314	0.04%	0.03%	0.05%
**Study sites:**							
Kabul	243	171	331	1,100,063	0.02%	0.02%	0.03%
Herat	29	23	35	141,226	0.02%	0.02%	0.02%
Mazar	48	38	60	120,343	0.04%	0.03%	0.05%
Jalalabad	28	22	35	67,769	0.04%	0.03%	0.05%
Kunduz	42	30	55	47,129	0.09%	0.06%	0.12%
Faizabad	14	11	18	9,721	0.14%	0.11%	0.19%
Kandahar	48	36	60	130,419	0.04%	0.03%	0.05%
Zaranj	32	25	39	7,617	0.42%	0.33%	0.51%
**Subtotal**	484	356	633	1,624,287	0.03%	0.02%	0.04%
**In 31 urban sites**	562	417	734	1,842,601	0.03%	0.02%	0.04%
**National (urban and rural)**	2,457	1,823	3,210	8,055,068	0.03%	0.02%	0.04%

**Table 10:** Extrapolated population size and prevalence estimates for male and female people who inject drugs (PWID) in the adult population (15–64 years old) in major cities in Afghanistan, and at the national level in 2019 (adjusted for winter seasonality effect).

City	Number of persons			Adult male and female population	Prevalence (%)		
	Point	Lower Bound	Upper Bound		Point	Lower Bound	Upper Bound
**Extrapolated sites:**							
Asadābād	29	21	38	8,204	0.35%	0.26%	0.46%
Aybak	54	39	73	17,490	0.31%	0.22%	0.42%
Bāmiyān	21	15	28	7,604	0.28%	0.20%	0.37%
Chaghcharān	23	17	29	4,181	0.55%	0.41%	0.69%
Chārīkār	99	72	133	33,980	0.29%	0.21%	0.39%
Farāh	73	53	96	22,032	0.33%	0.24%	0.44%
Gardīz	67	50	86	14,592	0.46%	0.34%	0.59%
Ghaznī	145	108	189	35,567	0.41%	0.30%	0.53%
Khowst	42	32	53	6,914	0.61%	0.46%	0.77%
Lashkar Gāh	236	177	302	46,091	0.51%	0.38%	0.66%
Maḥmūd-e Rāqī	3	3	4	908	0.33%	0.33%	0.44%
Maydān Shahr	9	7	12	1,826	0.49%	0.38%	0.66%
Mehtarlām	13	10	17	3,038	0.43%	0.33%	0.56%
Meymaneh	220	164	284	49,471	0.44%	0.33%	0.57%
Pol-e ’Alam	15	12	20	3,268	0.46%	0.37%	0.61%
Pol-e Khomrī	134	94	185	63,754	0.21%	0.15%	0.29%
Qalāt	27	20	35	6,706	0.40%	0.30%	0.52%
Qal’eh-ye Now	30	22	40	8,738	0.34%	0.25%	0.46%
Sar-e Pol	45	32	61	18,159	0.25%	0.18%	0.34%
Sharan	10	8	13	2,603	0.38%	0.31%	0.50%
Sheberghān	216	161	279	48,268	0.45%	0.33%	0.58%
Tāloqān	173	128	226	43,258	0.40%	0.30%	0.52%
Tarīn Kowt	21	16	27	4,029	0.52%	0.40%	0.67%
**Subtotal**	1,705	1,261	2,230	450,681	0.38%	0.28%	0.49%
**Study sites:**							
Kabul	6,062	4,258	8,255	2,225,687	0.27%	0.19%	0.37%
Herat	706	568	861	291,336	0.24%	0.19%	0.30%
Mazar	1,193	935	1,494	245,720	0.49%	0.38%	0.61%
Jalalabad	691	539	860	137,868	0.50%	0.39%	0.62%
Kunduz	1,031	740	1,362	96,000	1.07%	0.77%	1.42%
Faizabad	343	259	435	19,910	1.72%	1.30%	2.18%
Kandahar	1,177	897	1,485	265,355	0.44%	0.34%	0.56%
Zaranj	787	609	974	15,656	5.03%	3.89%	6.22%
**Subtotal**	11,990	8,805	15,726	3,297,532	0.36%	0.27%	0.48%
**In 31 urban sites**	13,695	10,066	17,956	3,748,213	0.37%	0.27%	0.48%
**National (urban and rural)**	57,207	42,049	75,005	16,003,853	0.37%	0.27%	0.48%

### Extrapolation of population sizes to other cities

Using proxy variables, we extrapolated the number of male PWID to the 23 other largest cities in Afghanistan which correspond to the provincial capitals, and then, arrived at the national adult population estimate of PWID (Table 8). The male-to-total ratios extrapolated the female PWID population sizes (Table 9), and the total number of PWID (Table 10). The total number of male PWID in Afghanistan was estimated to be 54,782 (95% CI 40,250–71,837), corresponding to 0.69% (95% CI 0.51–0.90) of the adult male population age 15 to 64 years. The total number of female PWID in Afghanistan was estimated to be 2,457 (95% CI 1,823–3,210), corresponding to 0.03% (95% CI 0.02–0.04) of the adult female population age 15 to 64 years. The total number of PWID in Afghanistan was estimated to be 57,207 (95% CI 42,049–75,005), which corresponds to 0.37% (95% CI 0.27–0.48) of the adult population age 15 to 64 years.

## Discussion

Applying and synthesizing different methods, our study projects 57,207 PWID in Afghanistan, corresponding to 0.37% of the population age 15 to 64 years. Given the estimated HIV prevalence of 4.4% among PWID [4], the number living with HIV would be 2,517. Between 2013 and 2019, HIV programs in Afghanistan diagnosed 371 (including only 22 in 2019) patients with HIV who reported drug injection as the mode of transmission [19] indicating a large number of PWID living with HIV who are undiagnosed. As there is no standard method to estimate the number of hidden populations, we used several different methods in this study. To our knowledge, this is the first study that used multiple methods and a Bayesian synthesis of results in the Eastern Mediterranean region countries. A principal finding of our study is the large gap in HIV testing services reaching this key population in Afghanistan. To reach to the UNAIDS 90-90-90 targets, the PWID population needs to be prioritized for increased testing, which is also the entrée to treatment programs.

Our estimate for the proportion of PWID injecting in the last 12 months in the national population is higher than the estimation of PWID in two neighbor countries, Pakistan and Iran. In Pakistan in 2010, the proportion of PWID in the population 15–64 years was estimated at 0.14% [20]. In Iran, the national prevalence for PWID was estimated at 0.28% in 2013 [21]. Our Afghanistan estimated proportion of PWID was lower than the estimated proportion of PWID in Tajikistan and Uzbekistan (0.45% and 0.47%, respectively) [22]. Of note, methods used in the countries varied. For example, the size estimation conducted in Iran used the network scale-up method to estimate the number of PWID [21].

We found the estimated number of PWID was many fold higher among males compared to females. We found a 22:1 ratio of male to female PWID (0.69% in male versus 0.03% in female). This ratio compares to 2 (0.59% in male versus 0.28% in female) in Australia [23], and 12.1 (193,000 male versus 16,000 female) [21] in Iran. We also found the number of female PWID at hotspots and public venues to be very low to nil. In many countries in the region, stigma around drug use and injection for women is very high which likely led to very few female PWID attending in public hotspots. Drug use stigma may also reduce substance use related health services access and utilization for women [24].

We found that more than half (57%) of the PWID in the hotspots were younger than 35 years. In Iran, the average age of PWID in a systematic review of 21 studies ranged from 28.8 to 39.8 years [25]. The majority of PWID in developed countries, such as Australia [23], are reported to be older than 35 years. Mateu-Gelabert et al reported that young drug users tend to interact with older drug users who have a higher prevalence of HIV or HCV [26], putting the younger PWID at elevated risk of these blood-borne infections. Assuming the same pattern in Afghanistan, the young age of the PWID population in Afghanistan presents an opportunity for harm reduction and other prevention programs to prevent further transmission of HIV and HCV.

There was a notably high median number of PWID observed in public hotspots (~11). Public hotspots in Afghanistan were crowded, suggesting possible large networks of PWID increasing the risk of HIV and other blood-borne transmission. Methodologically, our study expanded the reverse tracking method from its application for an overall method to one that can estimate the population of PWID in each hotspot [9]. Accessing PWID through outreach programs, such as mobile testing and harm reduction services [27], with frequent visiting of hotspots may prove to be effective strategy, particularly for those who do not visit facilities. Harm reduction service for people who use or inject drugs started in 2007 in Afghanistan and provided services in 12 provinces. While these centers provided free harm reduction services (e.g., needle and syringe exchange, condom distribution, referral to methadone clinics), more work is needed to improve the access and the utilization of these programs in the country. According to program data from Afghanistan National Program for Control of AIDS, STI and Hepatitis (personal communication, July 20, 2020), during 2019 a total of 8,265 PWID were reached by the harm reduction programs, with 1,228 referred to opioid substitution therapy, 2,893,605 sterile needles and syringes distributed, and 271,933 condoms given to PWID. Given the total population size of PWID estimated for Afghanistan, the coverage of harm reduction program is about 14%, the coverage of opioid substitution is about 2%, and per each PWID 50 sterile needles and syringes and 5 condoms were distributed in 2019. The results of our study can help set realistic targets, direct programs to particular hotspots, and evaluate their reach. The policy makers and public health administrators should use the results of this study for planning a better harm reduction program in Afghanistan.

One of the methods that we used for estimating the PWID population size was capture-recapture. We noted the potential impact of violating the assumption of independence for capture-recapture. We modeled and reduced potential bias resulting from non-independence of capture occasions [28] by log-linear models. The independence assumption assumes that being ‘captured’ (observed) on one capture occasion (e.g., in our study, being given a unique object) does not increase or decrease one’s probability of being ‘captured (observed) on another capture occasion (e.g., in our study, participating in the first or second interview). If there is a positive dependence between capture occasions (people observed on one capture occasion are also more likely to be observed on another capture occasion), the unobserved population size will be under-estimated. If there is negative dependence between capture occasions (people observed on one capture occasion are less likely to be observed on another capture occasion), the unobserved population size will be over-estimated. It is common to use interaction terms in a log-linear regression model to correct the potential bias created by non-independence of capture occasions. The interaction terms represent the non-independence of two capture occasions. With three capture occasions, as we had in our study, eight different log-linear models were possible, each with different combinations of pairwise interactions terms, reflecting non-independence of the capture occasions (e.g., unique object and first interview, unique object and second interview, first interview and second interview). Traditionally, the model with the lowest AIC is selected as the best-fitting model. The closed population assumption assumes that there are no entries or exits to the population during the capture-recapture study [29]. We worked to meet this assumption by design; participants were asked about their intravenous drug use over the past year, and the three separate capture occasions all took place over a short period of time (within two months of each other). This limits the likelihood that someone was an active member of the population during the first capture (the unique object) but not the last capture two months later (the second interview), or vice versa. If this assumption were not met, it would result in reducing the potential overlap in capture occasions, which would likely over-estimate the population.

Our study had several limitations. First, although we used trained staff who were supported by peers (current or past drug users) to recognize, encounter, and engage PWID in hotspots, our team may have counted people who were not PWID. Second, we did not visit and enumerate the number of PWID inside private locations (e.g., in homes, private buildings) due to security risks to study staff and the target population. This may have led to underestimation of PWID. Nonetheless, our estimation methods project counts for those who could be reached by programs. Third, some PWID could be counted in more than one hotspot if they were present in different locations at the time of survey. This would overestimate the number of PWID. To reduce this limitation, we did the survey in a short time period of 3–4 weeks in each city. Fourth, our estimates were from a cross-sectional survey, measured at one point in time, and were therefore vulnerable to temporal and seasonal effects. These effects could change the estimated number of PWID who visited the hotspot. We partly addressed the seasonality pattern by using the monthly variation in the number of PWID who visited DIC to adjust results for the winter seasonality effect. Fifth, due to stigma and social desirability, some PWID may avoid hotspots or not disclose their injection behavior. This could have led to underestimating the number of PWID. Sixth, our extrapolation results for other cities in Afghanistan should be considered with caution as we used a model assuming indicators correlated with drug injection behaviors in eight large cities will project the population size of PWID across all large and small cities. For example, the proportion of PWID in smaller towns may be much lower than the proportion in the big cities, therefore the extrapolation may overestimate the number of PWID in Afghanistan.

Despite limitations, we were able to directly find the locations and estimate number of PWID in hotspots and several cities in Afghanistan. These estimates can be used by local and national stakeholders for better planning and resource allocation. We estimated the number of PWID to be less than 4 persons per 1000 adult population. We also identified many hotspots that can be used to reach PWID for prevention and harm reduction services. The young demographic of the majority of PWID (over half under the age of 35) calls for better strategies to prevent acquiring blood-borne infections and harm associated with drug injection before greater morbidity and mortality occur. As there is no gold standard for size estimation in practice, we are unable to recommend using only one of the methods deployed in the current study. We recommend that size estimation be conducted by different approaches and results synthesized, such as by the Bayesian approach we provide.

## Supporting information

S1 Appendix Forms and questionnaires in English for mapping and population size estimation of PWID in Afghanistan. (ZIP)

S2 Appendix Forms and questionnaires in Dari for mapping and population size estimation of PWID in Afghanistan. (ZIP)

S3 Appendix Forms and questionnaires in Pashtoo for mapping and population size estimation of PWID in Afghanistan. (ZIP)

S4 Appendix Unduplicated counts of people who inject drugs (PWID) reached by specific programs in each city, Afghanistan. (DOCX)

S5 Appendix Proxy indicators to extrapolate the population size estimations for people who inject drugs (PWID) from study sites to non-study sites, Afghanistan, 2019. (DOCX)
