# Supplementary material for: Mapping and population size estimates of people who inject drugs in Afghanistan in 2019: Synthesis of multiple methods
Source: PLoS One. 2022 Jan 28;17(1):e0262405. doi: 10.1371/journal.pone.0262405 (PMC8797259; doi:10.1371/journal.pone.0262405)
Supplement: S1 Appendix — (ZIP) [file pone.0262405.s001.zip › PWID-English Tools/Appendix 11.docx]

### Appendix 11. Service data collection matrix from NGO and public providers

This form is used to count the number individual clients and types of services for use in the population size estimation multiplier calculations.

**Table 1: number of individual persons who inject drugs (PWID) visited**

| Name of NGO/ Facility and  address | Type of service being provided [choose only one per row] | Number of induvial persons of PWID who have been visited [exclude duplicates] | The time period for the reported count [Example: Jan to June 2018] | Data manager name, email and phone number |
| --- | --- | --- | --- | --- |
|  | ☐ Drop in Centre (DIC) ☐ HIV test ☐ Free sterile needles/syringes  ☐ Free condom  ☐ STIs test or treatment  ☐ Drug treatment (MMT)  ☐ Hospitalization  ☐ Shelter  ☐ Arrest  ☐ Other (specify clearly ______________________________) |  |  |  |
|  | ☐ Drop in Centre (DIC) ☐ HIV test ☐ Free sterile needles/syringes ☐ Free condom  ☐ STI test or treatment  ☐ Drug treatment (MMT)  ☐ Hospitalization  ☐ Shelter  ☐ Arrest  ☐ Other (specify clearly ______________________________) |  |  |  |

**Table 2: number of individual men who have sex with other men (MHRB) visited**

| Name of NGO/ Facility and  address | Type of service being provided [choose only one per row] | Number of individual persons of MHRB who have been visited [exclude duplicates] | The time period for the reported count [Example: Jan to June 2018] | Data manager name, email and phone number |
| --- | --- | --- | --- | --- |
|  | ☐ HIV test ☐ Free condom  ☐ STIs test or treatment  ☐ Hospitalization  ☐ Shelter  ☐ Arrest  ☐ Other (specify clearly ______________________________) |  |  |  |
|  | ☐ HIV test ☐ Free condom  ☐ STIs test or treatment  ☐ Hospitalization  ☐ Shelter  ☐ Arrest  ☐ Other (specify clearly ______________________________) |  |  |  |
|  | ☐ HIV test ☐ Free condom  ☐ STIs test or treatment  ☐ Hospitalization  ☐ Shelter  ☐ Arrest  ☐ Other (specify clearly ______________________________) |  |  |  |

**Table 3: number of individual women who have sold sex for money, drugs, or goods (WHRB) visited**

| Name of NGO/ Facility and address | Type of service being provided [choose only one per row] | Number of individual persons of WHRB who have been visited [exclude duplicates] | The time period for the reported count [Example: Jan to June 2018] | Data manager name, email and phone number |
| --- | --- | --- | --- | --- |
|  | ☐ HIV test ☐ Free condom  ☐ STIs test or treatment  ☐ Hospitalization  ☐ Shelter  ☐ Arrest  ☐ Other (specify clearly ______________________________) |  |  |  |
|  | ☐ HIV test ☐ Free condom  ☐ STIs test or treatment  ☐ Hospitalization  ☐ Shelter  ☐ Arrest  ☐ Other (specify clearly ______________________________) |  |  |  |
|  | ☐ HIV test ☐ Free condom  ☐ STIs test or treatment  ☐ Hospitalization  ☐ Shelter  ☐ Arrest  ☐ Other (specify clearly ______________________________) |  |  |  |
