# Supplementary material for: Mapping and population size estimates of people who inject drugs in Afghanistan in 2019: Synthesis of multiple methods
Source: PLoS One. 2022 Jan 28;17(1):e0262405. doi: 10.1371/journal.pone.0262405 (PMC8797259; doi:10.1371/journal.pone.0262405)
Supplement: S1 Appendix — (ZIP) [file pone.0262405.s001.zip › PWID-English Tools/Appendix 13.docx]

### Appendix 13. Oral Consent Form for interview with key informants and NGO staff serving the key populations

**Project Title:** Population Size Estimation and Mapping of Three Key Populations in Afghanistan in 2019

**Introduction and Purpose**

People who use drugs or have sexual risk behaviors are often at increased risk for health problems, including HIV. In collaboration with the Ministry of Health Afghanistan, we conducting an assessment to learn more about where these populations can be reached throughout the eight cities in Afghanistan. The information collected during this study will be used to target prevention interventions with these populations.

**Procedures**

We will ask you to do an individual interview. The interview is voluntary, no identifying information will be recorded, and we will do all efforts to keep what we discuss confidential. The interview will take about 30 minutes of your time. If you agree to be in the study, we will ask you some questions related to your knowledge of people with risk behaviors in this hotspot and also in other hotspots in the town. Some examples of questions that we will ask include:

- Locations of hotspots where key populations frequent
- Your estimate of the number of people with high risk behaviors who frequent these hotspots
- Availability of and access to health services and other services targeting these populations, such as sober houses

We will take notes during the interview. The notes will be used to help us keep an accurate record of what is said during the interviews for when we write the report.

**Benefits**

You may not benefit directly from being in the study. However, you or someone you know may benefit indirectly because what we learn will help us to reach and improve services to most who needed.

**Risks or Discomforts**

There is a slight risk that you could be identified by your role and the kind of work that you do. It is not possible to absolutely guarantee that you could not be identified. We will not use your name, and we will take great care to protect your privacy.

**Confidentiality**

We will not record or use your name or any other identifying information. We will code your interview with a number to help us keep track of our notes from the interview. There will be no link to your name at any time. We will destroy the notes after the study is done.

All study staff have signed confidentiality agreements. Study staff will not discuss what they learn or hear during interviews with your employer or anyone else outside of the study team.

**Cost/Payment**

There is no cost to you for being in the study.

**Compensation**

Your participation is voluntary. We will not be providing any compensation.

**Right to Refuse or Withdraw**

You are free to choose to not be in the study. If you choose not to be in the study, there is no penalty. If you decide to be in the study, you are free to stop at any time without any penalty. You do not have to give us a reason for stopping.

**Persons to Contact**

If you have questions about the study or believe that you have been harmed by being in the study, you may call Dr. Hamdard Naqibullah, Head of Afghanistan's National Program for Control of AIDS, Hepatitis & STI-ANPASH, Ministry of Public Health-MoPH 0093-795590772

If you have questions about your rights as a participant or report violations, please contact: Dr. Abdul Rasheed, Project County Director, Youth Health and Development Organization (YHDO), House No.63, Street No.2, Karte 3, District 6, Kabul, Afghanistan, Mobile# +93 (0) 700 072 109

Do you have any questions about what I have just said?
