# Supplementary material for: Mapping and population size estimates of people who inject drugs in Afghanistan in 2019: Synthesis of multiple methods
Source: PLoS One. 2022 Jan 28;17(1):e0262405. doi: 10.1371/journal.pone.0262405 (PMC8797259; doi:10.1371/journal.pone.0262405)
Supplement: S1 Appendix — (ZIP) [file pone.0262405.s001.zip › PWID-English Tools/Appendix 15.docx]

### Appendix 15. Consent Form for Focus Group Discussion

Consent Form for Focus Group Discussion

Youth Health and Development Organization (YHDO) invites you to be part of this study. The information I will give you can help you make an informed choice about joining the study.

1. Why we are doing this study?

People who use drugs or have sexual risk behaviors are often at increased risk for health problems, including HIV. In collaboration with the Ministry of Health Afghanistan, we conducting an assessment to learn more about where these populations can be reached throughout the eight cities in Afghanistan. The information collected during this study will be used to target prevention interventions with these populations.

1. What will happen?

If you agree to be in this study, this is what will happen.

- You will be asked to participate in a group discussion with about 6-10 other people to talk about locations of hot spots where drug use and sex work take place in this city and also your estimated number of these populations.
- We will have 2 study staff present during the discussion. One of them will be taking notes on paper or on a computer about what is being said.
- We would like to audio-record the discussion, so we can listen to it later, and make sure we don’t miss any comments. If you do not want us to audio-record the discussion, please let us know, and we will only take notes. After transcribing the discussions from the audio-tape, we will destroy the recording.
- To protect your confidentiality, we will ask you not to use your full name during the group discussion; you can use your first name or a name that is not your own, if you prefer.

1. Where will this happen.

The discussion will take place at our study site in Kabul or in our collaborating sites in other cities in Afghanistan.

1. Study funder, collaborators and implementer.

The study has been funded by UNDP/Global Fund Program. Experts from Ministry of Health, and University of California San Francisco, and Kerman University of Medical Sciences have designed the study and will be analyzing the data gathered. YHDO implements, collects data and monitors the study progress as specified in the study protocol.

1. How many people will be in the study. In total, about 30 people will participate in focus group discussions in each city.
2. How long will this part of the study take?

The group discussion will last a maximum of 1.5 hours.

1. Things to consider and possible risks

There are some risks from being in this study:

- You may feel discomfort talking about drug use or sex wok in your city.
- You and all focus group participants are asked to keep what is said in the group private, but there is some risk that group members may talk about what was said to other people who did not participate in the group.

1. Confidentiality

We will do everything we can to protect your confidentiality. Your responses will be labeled with a study number only, and we will not ask you your name, address, phone number or other identifying information. We will ask you not to use your full name during the discussion group, and we will not ask for any of your personal information.

1. Benefits

You may not benefit directly from being in the study. However, you or someone you know may benefit indirectly because what we learn will help us to reach and improve services to most who needed.

1. Alternatives

You are free to choose not to participate in the study without any penalty. This study is completely voluntary.

1. Compensation

Participants will be provided a meal and reimbursement for their travel expenses.

1. Persons to contact for any questions about the study

If you have questions about the study or believe that you have been harmed by being in the study, you may call Dr. HamdardNaqibullah, Head of Afghanistan's National Program for Control of AIDS, Hepatitis & STI-ANPASH, Ministry of Public Health-MoPH 0093-795590772

If you have questions about your rights as a participant or report violations, please contact: Dr. Abdul Rasheed, Project County Director, Youth Health and Development Organization (YHDO), House No.63, Street No.2, Karte 3, District 6, Kabul, Afghanistan, Mobile# +93 (0) 700 072 109

1. Costs. There will be no costs to you for taking part in this study.
2. Agreement

Do you have any questions?

Participation in study is VOLUNTARY. If you don’t wish to participate, you are free to leave now.If you want to participate, please stay here and we will do the focus group discussion.
