# Supplementary material for: Mapping and population size estimates of people who inject drugs in Afghanistan in 2019: Synthesis of multiple methods
Source: PLoS One. 2022 Jan 28;17(1):e0262405. doi: 10.1371/journal.pone.0262405 (PMC8797259; doi:10.1371/journal.pone.0262405)
Supplement: S1 Appendix — (ZIP) [file pone.0262405.s001.zip › PWID-English Tools/Appendix 22- Supervision form.docx]

### Appendix 22. Supervision form

The City Supervisor completes this form whenever they meet with the data collection teams either in office for morning sessions or at the hotspots.

Population: PWID WHRB MHRB

Date: …….…/…….…/…….… Data Collection team ID: ……………… City: …………………

| If this form is being completed for specific hotspot(s), write the IDs and addresses: |
| --- |
| Hotspot(s) IDs: ……………………………………………………………………..…………………………………………………..…..….  Hotspot(s) Address: …………………..…………………………………………………………………………..………………………… |

1. **Field manger assessment**

| 1. Team visited the hotspot at the scheduled date/time? | Yes No Not assessed |
| --- | --- |
| 1. Security measures were assessed and managed carefully? | Yes No Not assessed |
| 1. Any violence or events reported or observed? | Yes No Not assessed |
| 1. Enumeration form did not miss any key information? | Yes No Not assessed |
| 1. GPS code recorded correctly? | Yes No Not assessed |
| 1. Number of interviews with member of key populations was 2 to 8? | Yes No Not assessed |
| 1. Number of interviews with other key informants was 3 to 5? | Yes No Not assessed |
| 1. Duration of presence at the hotspot was minimum 2 hours? | Yes No Not assessed |

**If the answer to any of the above questions is No, investigate for the reasons and troubleshoot. Write bellow the reasons and the action you did for troubleshooting:**

………………………………………………………………………………………………………………………………………………………………………………………………………………………………………………………………………………………………………………………………………………………………………………………………………………………………………………………………………………………………………………………………………………………………………………………………………………………………………………………………………………………………………..

1. **Interviewer assessment**

| 1. Screening for eligibility was done appropriately? | Yes No Not assessed |
| --- | --- |
| 1. Only eligible people were enrolled? | Yes No Not assessed |
| 1. Response rate (% eligible people who interviewed) was more than 80%? | Yes No Not assessed |
| 1. Collecting informed consents were done correctly? | Yes No Not assessed |
| 1. A private spot used for interviews? | Yes No Not assessed |
| 1. Zero missing data for key questions? | Yes No Not assessed |
| 1. Consistency between data from different sections and questions? | Yes No Not assessed |
| 1. Incentive was given at the end and appropriately? | Yes No Not assessed |

**If the answer to any of the above questions is No, investigate for the reasons and troubleshoot. Write bellow the reasons and the action you did for troubleshooting:**

…………………………………………………………………………………………………………………………………………………………………………………………………………………………………………………………………………………………………………………………………………………………………………………………………………………………………………………………………………………………………………………………………………………………………………………………………………………………………………………………………………………………………………
