# Supplementary material for: Mapping and population size estimates of people who inject drugs in Afghanistan in 2019: Synthesis of multiple methods
Source: PLoS One. 2022 Jan 28;17(1):e0262405. doi: 10.1371/journal.pone.0262405 (PMC8797259; doi:10.1371/journal.pone.0262405)
Supplement: S1 Appendix — (ZIP) [file pone.0262405.s001.zip › PWID-English Tools/Appendix 3.docx]

### Appendix 3. Key Informant Interview and FGD guide for PWID

**Key Informant Interview and FGD guide**

**Mapping of Injecting Drug Users in Afghanistan**

**Interview ID: ____________________________________**

**Interviewer/FGD leader name: ____________________________________**

**Note taker ID: ____________________________________**

**Note taker name: ____________________________________**

**Date of interview/FGD (mm/dd/yy): ___________________________**

**[For FGD, collect the FGD oral consent]**

**[For interview, collect the oral consent for key informants and NGO staff servicing the key populations]**

**Introduction**

Thank you for agreeing to participate in this interview. Remember that your answers will be kept confidential and you can stop the interview at any time. Please do not use your name or the names of any acquaintances. You may use initials or pseudonyms.

**ASK ALL INFORMANTS**

1. Please tell me about the districts where drug activity use taking place in ______________ (name of city)? Including selling, using, injecting, hanging out. *If not specifically mentioned probe about injection drug use.*

[**Use a paper or digital map with clear districts borders to guide the discussion**]

1. Now I’d like your help making a list of each of the hotspots by districts. [create 5 column table, with hotspot names in 1^st^ column]
   1. What sort of hotspots are each of these hotspots in these different districts? [populate 2^nd^ column with location/type. This helps locate the types of places and the spatial distances from them. Example: behind the main market/abandoned building]
   2. Please tell me how many drug users (including those who also inject drugs) – male and female – you would estimate hang out in each of these sites you just mentioned on a typical day? [populate 3^rd^ column]
   3. Please tell me how many drug injectors – male and female – you would estimate hang out in each of these sites you just mentioned on a typical day? [populate 4^th^ column]
   4. Please tell me how many heroin injectors you would estimate hang out in each of these hotspots you just mentioned on a typical day? And how many of these are women? [populate 5^th^ column]
   5. Please tell me the day and time when drug users/injectors visit the hotspot [populate 6^th^ column]
   6. Please tell me the day and time when the highest number of drug users/injectors visit the hotspot (peak day/time) [populate 7^th^ column]

| **1** | **2** | **3** | | | **4** | | **5** | | **6** | **7** |
| --- | --- | --- | --- | --- | --- | --- | --- | --- | --- | --- |
| District | Hotspot location/type | # of all users | | | # of drug injectors | | # of heroin injectors | | All working days/times | Peak day/time |
|  |  | M | F | M | | F | M | F |  |  |
|  |  |  |  |  | |  |  |  |  |  |
|  |  |  |  |  | |  |  |  |  |  |
|  |  |  |  |  | |  |  |  |  |  |
|  |  |  |  |  | |  |  |  |  |  |
|  |  |  |  |  | |  |  |  |  |  |
|  |  |  |  |  | |  |  |  |  |  |

**ASK NGOs/SERVICE PROVIDERS**

1. Please describe the interaction your organization has with drug users and injectors – what services do you provide them, if any?
2. How many individual drug users and injectors access your services each month?
3. How do you reach drug users and injectors?
4. What do you think the barriers are for drug users or injectors to access health services?
5. Are you aware of any other organizations offering health or other services to drug users or injectors?
6. What health services are drug users most in need of?

**ASK PRIMARY KEY INFORMANTS WHO ARE PWID**

1. What services for drug users are you aware of in this area? Probe: HIV Testing, Hepatitis Testing/Vaccination, Methadone, Needle exchange, drug treatment, legal services.
2. Do you use those services? Which ones? How often? Are you able to get clean syringes/needles when you need them? If not, why not? If so, where?
3. What types of services do you think are lacking/needed?
4. What do you think the barriers are for you to access health services?
5. How are drug users treated by people in this city? Probe: How are injection drug users treated in this city?
