# Supplementary material for: Mapping and population size estimates of people who inject drugs in Afghanistan in 2019: Synthesis of multiple methods
Source: PLoS One. 2022 Jan 28;17(1):e0262405. doi: 10.1371/journal.pone.0262405 (PMC8797259; doi:10.1371/journal.pone.0262405)
Supplement: S1 Appendix — (ZIP) [file pone.0262405.s001.zip › PWID-English Tools/Appendix 6.docx]

### Appendix 6. Hotspot Observation and Enumeration Form

| City name ___________ | **Field manager name** ___________ |
| --- | --- |
| Hotspot ID ___________ | **Data collector name** ___________ |
| Hotspot address (**number, street, cross street):**  ______________________________________________________________________ | **Date form completed** (dd/mm/yy): _______ |
|  | **Visit starting time** (24h clock, hh:mm): ________ |
|  | **Visit ending time** (24h clock, hh:mm): ________ |
|  | **Target Population**:  ☐ PWID☐ MHRB☐ WHRB |
|  | **Hotspot visit:** ☐ first visit☐ second visit |

**Hotspot GPS Coordinates**

| Longitude ______________ | Latitude ______________ |
| --- | --- |
| Mobile GPS code ______________ |  |

**Type of hotspot:**

☐ abandoned building ☐ park ☐ street location ☐ café or restaurant

☐ shopping center ☐ transportation terminal or hub ☐ inn/hotel ☐ private home
☐ others (describe bellow)

**Brief description of the hotspot:**

**_____________________________________________________________________________________
_____________________________________________________________________________________**

**_____________________________________________________________________________________
_____________________________________________________________________________________
_____________________________________________________________________________________
_____________________________________________________________________________________**

**Individuals counted through direct count**

| **Population** | **# Total** | **By sex group** | | **By age groups** | |
| --- | --- | --- | --- | --- | --- |
|  |  | **# Male** | **# Female** | **# <25 years old** | **# ≥25 years old** |
| PWID |  |  |  |  |  |
| MHRB | NA |  | NA |  |  |
| WHRB | NA | NA |  |  |  |

NA: Not Applicable

**Individuals approached for the short questionnaire**

| **Population** | **# approached** | **# refused to participate** |
| --- | --- | --- |
| PWID |  |  |
| MHRB |  |  |
| WHRB |  |  |

**Observed activities in the hotspot:**

☐looking for sex partner/clients ☐drug dealing ☐using drugs ☐injecting drugs

☐having sex ☐ a place to live ☐socialize ☐others (describe bellow)

**Brief description of other activities in the hotspot:**

**_____________________________________________________________________________________
_____________________________________________________________________________________**

**_____________________________________________________________________________________
_____________________________________________________________________________________
_____________________________________________________________________________________
_____________________________________________________________________________________
_____________________________________________________________________________________
_____________________________________________________________________________________**

**Observed signs in the hotspot:**

☐syringe/needle ☐other drug use equipment ☐ condoms ☐ other, specify:

**Were you able to take a few photos of the hotspot?** ☐yes ☐no

**Was it the time when the hotspot has the highest number of target population?**

☐yes ☐no

**Other comments or issues during the exercise:**

**_____________________________________________________________________________________
_____________________________________________________________________________________**

**_____________________________________________________________________________________
_____________________________________________________________________________________**

**_____________________________________________________________________________________
_____________________________________________________________________________________**

**_____________________________________________________________________________________
_____________________________________________________________________________________**

**_____________________________________________________________________________________
_____________________________________________________________________________________
_____________________________________________________________________________________
_____________________________________________________________________________________
_____________________________________________________________________________________**
