# Supplementary material for: Mapping and population size estimates of people who inject drugs in Afghanistan in 2019: Synthesis of multiple methods
Source: PLoS One. 2022 Jan 28;17(1):e0262405. doi: 10.1371/journal.pone.0262405 (PMC8797259; doi:10.1371/journal.pone.0262405)
Supplement: S1 Appendix — (ZIP) [file pone.0262405.s001.zip › PWID-English Tools/Appendix 7.docx]

### Appendix 7. PWID Screening and Data Collection Form

We are conducting a study to understand the best locations where we can reach people for prevention services. To participate in this project, we must first ask you a few questions. Your answers will tell us if you are eligible to participate in this study. If you are eligible, we will review the consent form with you. Your answers to all questions will remain private, meaning we will not share it with anyone. If you are found to be eligible, we will ask you to participate in the study. The study takes less than 10 minutes of your time and you will be paid (amount equal to 1$) for the time you spend taking part in the study.

**[Eligibility Questions]**

| **Question** | **Response** |
| --- | --- |
| 1. In which languages are you fluent? (must be fluent in at least one of the following languages to be eligible for the study) | Pashtoo  Dari  Uzbek  Other **[stop screening, individual is not eligible]** |
| 1. How old are you? | _____________ [Write-in response. If 17 or younger, stop screening, individual is not eligible] |
| 1. Have you used any drugs within the last 12 months? | Yes **[continue to Q4]**  No **[stop screening, individual is ineligible]** |
| 1. Have you injected any drugs within the last 12 months? | Yes **[continue to Q5]**  No **[stop screening, individual is ineligible]** |
| 1. Have you participated in a survey like this in the past 3 months? | Yes **[stop screening, individual is ineligible]**  No **[continue to Demographic and drug related questions]** |

**[If the individual is eligible review and obtain verbal consent.]**

**Demographic and drug related questions**

| **Q#** | **Question** | **Response** | **Code** |
| --- | --- | --- | --- |
|  | Sex? | Male  Female  Other, please specify _________________ | 1  2  3 |
|  | When did you last inject any drugs? | Within the last 1 month  Within the last 3 months  Within the last 12 months | 1  2  3 |
|  | What drug(s) did you inject frequently?  **Check all that apply.** | Heroin  Cocaine  Opium  Amphetamines  Prescription drugs  Other_____________________ | 1  2  3  4  5  6 |

**Questions for population size estimation**

| **Q#** | **Question** | **Response** | **Code** |
| --- | --- | --- | --- |
|  | Did you participate in a study where you were asked questions like these, received HIV testing, and were given coupons to recruit your friends in 2012? | Yes  No  Not sure | 1  2  3 |
|  | Did you receive <unique object> in the past 3 months? | Yes  No | 1  2 |
|  | Did you receive <service a> from <center a> in the past 12 months? | Yes  No | 1  2 |
|  | Did you receive <service b> from <center b> in the past 12 months? | Yes  No | 1  2 |
|  | How many other people who **inject** drugs do you think are living in <city>?  *Probe: if the number is very small or very big (e.g. more than the population living in the city), then explain to the person and ask to rethink. Also, number of Male and Female should add up to total.* | **Total:** Minimum _______ Maximum _______  **Male:** Minimum _______ Maximum _______  **Female:** Minimum _______ Maximum _______ | ---- |

**Questions about the current hotspot**

| **Q#** | **Question** | **Response** | **Code** |
| --- | --- | --- | --- |
|  | In the past 30 days, how often did you attend this hotspot? | 1 – 5 times  6 – 10 times  11 – 15 times  16 – 20 times  21 – 25 times  26+ times  Do not know  Refuse to answer | 1  2  3  4  5  6  7  8 |
|  | When does this hotspot have the highest number of people who use or inject drugs? | ________ Day  ________ Time of day  _________Specific date (dd/mm/yy) | ---- |
|  | How many different people who **inject** drugs have you seen in this hotspot in the **past week**? | ______ Male  ______Female | ---- |
|  | How many different people who **inject** drugs have you seen in this hotspot in the **past month**? | ______ Male  ______Female | ---- |
|  | Which of the following services have been provided at this hotspot during the past month? | Free needle or syringe  Free condom  Free HIV counseling and testing  Free education prevention materials  Others (please mention ______________________________________)  None as I know. | 1  2  3  4  5  6 |
|  | Did police sweeping happen in this hotspot during the past month | Yes  No  I do not know | 1  2  3 |

**Question about other three hotspots where the person visits frequently**

| **Q#** | **Question** | **Response** | **Code** |
| --- | --- | --- | --- |
|  | How many other hotspots do you go to most frequently to inject drugs or to meet with others who inject drugs? | I do not go to other hotspots (**skip to Q22**)  one hotspot  two hotspots  three hotspots  more than three (how many ________) | 1  2  3  4  5 |
|  | What is the name/address of the other hotspot you go to most frequently to inject drugs or to meet with others who inject drugs | Name _____________________________  Address ___________________________  __________________________________ | ---- |
|  | In the past 30 days, how often did you attend this hotspot? | 1 – 5 times  6 – 10 times  11 – 15 times  16 – 20 times  21 – 25 times  26+ times  Do not know  Refuse to answer  Not Applicable | 1  2  3  4  5  6  7  8  9 |
|  | What is the name/address of the second hotspot you go to most frequently to inject drugs or to meet with others who inject drugs? | Name _____________________________  Address ___________________________  __________________________________ | ---- |
|  | In the past 30 days, how often did you attend this hotspot? | 1 – 5 times  6 – 10 times  11 – 15 times  16 – 20 times  21 – 25 times  26+ times  Do not know  Refuse to answer  Not Applicable | 1  2  3  4  5  6  7  8  9 |
|  | What is the name/address of the third hotspot you go to most frequently to inject drugs or to meet with others who inject drugs? | Name _____________________________  Address ___________________________  __________________________________ | ---- |
|  | In the past 30 days, how often did you attend this hotspot? | 1 – 5 times  6 – 10 times  11 – 15 times  16 – 20 times  21 – 25 times  26+ times  Do not know  Refuse to answer  Not Applicable | 1  2  3  4  5  6  7  8  9 |

**Other behavioral questions**

|  | What is your current marital status? | Single  Married and living with my partner  Married but not living with my partner  Not married but living with my partner  Separated/divorced  Widowed | 1  2  3  4  5  6 |
| --- | --- | --- | --- |
|  | **(For males)** Have you ever had anal or oral sex with another man? | Yes  No **(skip to 27)** | 1  2 |
|  | **(For males)** Have you had anal or oral sex with another man in the past 12 months? | Yes  No | 1  2 |
|  | **(For males)** Have you exchanged (anal or oral) sex for money or drugs in the past 12 months?  *Probe: If no, is it never or not in past 12 months?* | Yes  No, I have exchanged sex for money or drugs, but not in the past 12 months  No, I have never exchanged sex for money or drugs | 1  2  3 |
|  | **(For females)** Have you exchanged (vaginal, anal, or oral) sex for money or drugs in the past 3 months?  *Probe: If no, is it never or not in past 3 months?* | Yes  No, I have exchanged sex for money or drugs, but not in the past 3 months  No | 1  2  3 |
|  | Have you ever been tested for HIV? | Yes  No **(skip to next section)** | 1  2 |
|  | When was your last HIV test? | Within the last 12 months  1-2 years ago  More than 2 years ago | 1  2  3 |
|  | Do you know your HIV status? | Yes  No **(skip to next section)** | 1  2 |
|  | If you are comfortable saying, what is your status? | HIV negative  HIV positive  Not comfortable saying | 1  2  3 |

**Closing section**

| Hotspot ID |  |
| --- | --- |
| Participant ID number |  |
| Interviewer name |  |
| Date form completed (dd/mm/yy) |  |
