# Supplementary material for: Mapping and population size estimates of people who inject drugs in Afghanistan in 2019: Synthesis of multiple methods
Source: PLoS One. 2022 Jan 28;17(1):e0262405. doi: 10.1371/journal.pone.0262405 (PMC8797259; doi:10.1371/journal.pone.0262405)
Supplement: S2 Appendix — (ZIP) [file pone.0262405.s002.zip › PWID-Dari Tools/Appendix 11- Service data collection matrix from NGO and public providers (6).docx]

| ضمیمه**۱۱.** فورم جمع اوری داتا از سازمان های دولتی وغیر دولتی (NGO) ارایه کنند گان خدمات این فورم برای شمارش تعداد مشتریان یا خدمات گیرنده گان و انواع خدمات برای استفاده در محاسبه ضرایب تخمینی (Multiplier Calculations) اندازه جمعیت استفاده می شود. | | | | |
| --- | --- | --- | --- | --- |
| جدول 1: تعداد افراد تزریقی مواد مخدر **(PWID)** که بازدید شده اند | | | | |
| نام، ایمیل ادرس و نمبر تلیفون مدیرداتا | مدت زمانی راپوردهی برای شمارش  (مثلا: از جنوری الی دسمبر ۲۰۱۸)  نوت: اگر مدت زمانی راپوردهی یا ارایه خدمات از یک سال کم باشد، همان مدت زمانی را نوشته کنید. | تعداد افرادی تزریقی مواد مخدر(PWID) که بازدید شده اند [تکراری شامل نیستند] | نوع سرویس یا خدمت ارائه شده  [تمام خدماتی را که در طول یک سال گذشته ارائه شده انتخاب کنید] | نام سازمان غیر دولتی (NGO)/مرکز و ادرس |
|  |  |  | ☐مشوره دهی(DIC) |  |
|  |  |  | ☐اچ آی وی تست |  |
|  |  |  | ☐مفت سوزن/سرنج تعقیم شده |  |
|  |  |  | ☐مفت کندم |  |
|  |  |  | ☐تست یا تداوی امراض مقاربتی |  |
|  |  |  | ☐تداوی متادون |  |
|  |  |  | ☐ بستر کردن در شفاخانه |  |
|  |  |  | ☐ سرپناه |  |
|  |  |  | ☐ بندی کردن |  |
|  |  |  | ☐دیگر (واضح مشخص کنید________) |  |
|  |  |  | ☐مشوره دهی(DIC) |  |
|  |  |  | ☐اچ آی وی تست |  |
|  |  |  | ☐مفت سوزن/سرنج تعقیم شده |  |
|  |  |  | ☐مفت کندم |  |
|  |  |  | ☐تست یا تداوی امراض مقاربتی |  |
|  |  |  | ☐تداوی متادون |  |
|  |  |  | ☐ بستر کردن در شفاخانه |  |
|  |  |  | ☐ سرپناه |  |
|  |  |  | ☐ بندی کردن |  |
|  |  |  | ☐دیگر (واضح مشخص کنید________) |  |

| جدول 2: تعداد مردان پرخطر(**MHRB**) که بازدید شده اند | | | | |
| --- | --- | --- | --- | --- |
| نام، ایمیل ادرس و نمبر تلیفون مدیرداتا | مدت زمانی راپوردهی برای شمارش  (مثلا: از جنوری الی دسمبر ۲۰۱۸)  نوت: اگر مدت زمانی راپوردهی یا ارایه خدمات از یک سال کم باشد، همان مدت زمانی را نوشته کنید. | تعداد افرادی مردان پرخطر(MHRB) که بازدید شده اند [تکراری شامل نیستند] | نوع سرویس یا خدمت ارائه شده  [تمام خدماتی را که در طول یک سال گذشته ارائه شده انتخاب کنید] | نام سازمان غیر دولتی (NGO)/مرکز و ادرس |
|  |  |  | ☐اچ آی وی تست |  |
|  |  |  | ☐مفت کندم |  |
|  |  |  | ☐تست یا تداوی امراض مقاربتی |  |
|  |  |  | ☐ بستر کردن در شفاخانه |  |
|  |  |  | ☐ سرپناه |  |
|  |  |  | ☐ بندی کردن |  |
|  |  |  | ☐دیگر (واضح مشخص کنید________) |  |
|  |  |  | ☐اچ آی وی تست |  |
|  |  |  | ☐مفت کندم |  |
|  |  |  | ☐تست یا تداوی امراض مقاربتی |  |
|  |  |  | ☐ بستر کردن در شفاخانه |  |
|  |  |  | ☐ سرپناه |  |
|  |  |  | ☐ بندی کردن |  |
|  |  |  | ☐دیگر (واضح مشخص کنید________) |  |

| جدول 3: تعداد زنان پرخطر(**WHRB**) که بازدید شده اند | | | | |
| --- | --- | --- | --- | --- |
| نام، ایمیل ادرس و نمبر تلیفون مدیرداتا | مدت زمانی راپوردهی برای شمارش  (مثلا: از جنوری الی دسمبر ۲۰۱۸)  نوت: اگر مدت زمانی راپوردهی یا ارایه خدمات از یک سال کم باشد، همان مدت زمانی را نوشته کنید. | تعداد افرادی زنان پرخطر(WHRB) که بازدید شده اند [تکراری شامل نیستند] | نوع سرویس یا خدمت ارائه شده  [تمام خدماتی را که در طول یک سال گذشته ارائه شده انتخاب کنید] | نام سازمان غیر دولتی (NGO)/مرکز و ادرس |
|  |  |  | ☐اچ آی وی تست |  |
|  |  |  | ☐مفت کندم |  |
|  |  |  | ☐تست یا تداوی امراض مقاربتی |  |
|  |  |  | ☐ بستر کردن در شفاخانه |  |
|  |  |  | ☐ سرپناه |  |
|  |  |  | ☐ بندی کردن |  |
|  |  |  | ☐دیگر (واضح مشخص کنید________) |  |
|  |  |  | ☐اچ آی وی تست |  |
|  |  |  | ☐مفت کندم |  |
|  |  |  | ☐تست یا تداوی امراض مقاربتی |  |
|  |  |  | ☐ بستر کردن در شفاخانه |  |
|  |  |  | ☐ سرپناه |  |
|  |  |  | ☐ بندی کردن |  |
|  |  |  | ☐دیگر (واضح مشخص کنید________) |  |
