# Supplementary material for: Mapping and population size estimates of people who inject drugs in Afghanistan in 2019: Synthesis of multiple methods
Source: PLoS One. 2022 Jan 28;17(1):e0262405. doi: 10.1371/journal.pone.0262405 (PMC8797259; doi:10.1371/journal.pone.0262405)
Supplement: S2 Appendix — (ZIP) [file pone.0262405.s002.zip › PWID-Dari Tools/Appendix 12. Oral Consent Form for interview from member of the key populations.docx]

# ضمیمه ۱۲: فورم رضایت نام شفاهی از اعضای جمعیت کلیدی برای مصاحبه

**عنوان پروژه: براورد اندازه ومکان یابی جمعیت های کلیدیدر هشت شهر افغانستان ۲۰۱۹**

**مقدمه و هدف**

افرادی که مواد را تزریق می کنند یا رفتارهای پر خطر جنسی دارد در معرض برخی مشکلات صحی مانند اچ آی وی قرار دارند. در همکاری با وزارت صحت عامه افغانستان، ما ارزیابی مینمایم تا بیشتربدانیم که جمعیت ها در کدام قسمت ها در هشت شهر افغانستان قابل دسترسی است. اطلاعات جمع آوری شده در مورد این مطالعه برای هدفگیری اقدامات پیشگیری با این جمعیت ها مورد استفاده قرار می گیرد.

**مصاحبه**

از شما خواهش میکنیم که یک مصاحبه انفرادی با ما انجام دهید. مصاحبه داوطلبانه میباشد و هیچ نوع اطلاعات شناسایی ثبت نمیشود و ما تمام تلاش خود را برای حفظ آنچه که ما در مورد محرمانه صحبت می کنیم، انجام می دهیم. مصاحبه حدود 10 دقیقه زمان شما را خواهد گرفت. اگر شما موافقت کردید که در این مطالعه شرکت کنید، از شما سوالاتی راجع به دانش شخصی تان در مورد افرادی که رفتارهای پرخطر دارند در این هات سپات و همچنین در هات سپات ها دیگر شهر یا منطقه خواهیم پرسید. برخی از نمونه سوالاتی که ما خواهیم پرسید این است:

- برخی سوالاتی درمورد خودتان مانند سن و رفتار های خطرناک مربوط استفاده مواد مخدر و روابط جنسی
- تخمین تعداد افرادی که رفتار های پرخطر دارند و مکررآ از این هات سپات بازدید میکنند.
- هات سپات های دیگری که در آن استفاده از مواد مخدریا روابط جنسی انجام می شود.
- بعضی سوالات در موړد سرویس های تست اچ آی وی که شما دریافت کرده اید.

ما از یک فورم برای نوشتن پاسخهای شما در مصاحبه استفاده خواهیم کرد.

**فواید**

شما ممکن است به طور مستقیم از مطالعه بهره مند نشوید؛ شما یا کسی که شما میشناسید ممکن است از این مطالعه به طور غیرمستقیم سود ببرد، زیرا آنچه که ما یاد میگیریم، به ما کمک میکند تا پیشنهادات خود را برای بهبود خدمات ارائه دهیم تا به نیازهای افرادی که ضرورت دارند برسند.

**خطرات یا ناراحتی**

یک خطر جزئی وجود دارد که شما می توانید با نقش و نوع کاری که انجام می دهید شناسایی شوید. این کاملا قطعی نیست که شما شناسایی نمیشوید. ما از نام شما استفاده نمی کنیم و کوشش زیادی خواهیم کرد تا از حریم خصوصی شما محافظت کنیم

**محرمانه بودن**

ما اسم و یا سایر اطلاعات شناسایی شما را ضبط نخواهیم کرد. ما مصاحبه شما را با یک عدد کود گذاری می کنیم تا به ما در ردیابی اطلاعاتمان از مصاحبه کمک کند. هیچ ارتباطی با نام شما در هر زمان وجود نخواهد داشت

کارکنان این مطالعه در مورد مصاحبه با کارفرمای شما و یا هر کسی که خارج از تیم تحقیق است، در مورد آنچه که آنها یاد می گیرند یا در جریان مصاحبه میشنوند صحبت نمی کنند. تصمیم شما راجع به شرکت یا عدم شرکت درمطالعه و همچنان پاسخ های شما بر هیچ یک از خدماتی که ممکن است شما دریافت کنید تأثیری نداشته باشد.

**هزینه / پرداخت**

برای اشتراک در مطالعه هزینه ای وجود ندارد.

**جبران خسارت**

ما در قبال وقت شما یک هدیه کوچک برابر ۷۵ افغانی خوا هیم داد.

**حق انتخاب با شما است**

شما می توانید انتخاب کنید که در مطالعه شرکت نخواهید کرد. اگر در مطالعه شرکت نمی کنید، مجازات نیست. اگر تصمیم گرفتید که در این مطالعه شرکت کنید، می توانید در هر زمان بدون مجازات توقف کنید. شما مجبور نیستید دلیل برای توقف داشته باشید

**افرادی که با انها تماس بیگرید.**

اگر در مورد این مطالعه سوالی دارید، یا باور دارید که به علت اشتراک در مطالعه برای شما کدام آسیب رسیده است، میتوانید با :

داکتر نقیب الله همدرد، ریس پروگرام ملی کنترول ایدز و هیپاتیت وزارت صحت عامه.

نمبر تلیفون: ۰۷۹۵۵۹۰۷۷۲ تماس بگیرید.

اگرشما در مورد حقوق شما به حیث اشتراک کننده سوال دارید یا میخواهید که نقض را گذرش دهد لطفا با:

داکتر عبدالرشید مسول پروژه در افغانستان وریس موسسه انکشافی و صحی برای جوانان

نمبر تلیفون: ۰۷۰۰۰۷۲۱۰۹ تماس بگیرید.

آیا در مورد آنچه که من گفتم، سؤالی دارید؟

آیاموافق هستید که در مصاحبه شرکت کنید؟
