# Supplementary material for: Mapping and population size estimates of people who inject drugs in Afghanistan in 2019: Synthesis of multiple methods
Source: PLoS One. 2022 Jan 28;17(1):e0262405. doi: 10.1371/journal.pone.0262405 (PMC8797259; doi:10.1371/journal.pone.0262405)
Supplement: S2 Appendix — (ZIP) [file pone.0262405.s002.zip › PWID-Dari Tools/Appendix 13. Oral Consent Form for interview with key informants and NGO staff serving the key populations.docx]

# ضمیمه ۱۳: فورم رضایت نامه شفاهی از معلومات دهنده کلیدی و کارکنان موسسات غیر دولتی که برای جمعی کلیدی خدمات عرضه میدارد.

**عنوان پروژه: براورد اندازه ومکان یابی جمعیت های کلیدی در هشت شهر افغانستان ۲۰۱۹**

**مقدمه و هدف**

افرادی که مواد را تزریق می کنند یا رفتارهای پر خطر جنسی دارد در معرض برخی مشکلات صحی مانند اچ آی وی قرار دارند. در همکاری با وزارت صحت عامه افغانستان، ما ارزیابی مینمایم تا بیشتربدانیم که جمعیت ها در کدام قسمت ها در هشت شهر افغانستان قابل دسترسی است. اطلاعات جمع آوری شده در مورد این مطالعه برای هدفگیری اقدامات پیشگیری با این جمعیت ها مورد استفاده قرار می گیرد.

**طرزالعمل**

از شما خواهش میکنیم که یک مصاحبه انفرادی با ما انجام دهید. مصاحبه داوطلبانه میباشد و هیچ نوع اطلاعات شناسایی ثبت نمیشود و ما تمام تلاش خود را برای حفظ آنچه که ما در مورد محرمانه صحبت می کنیم، انجام می دهیم. مصاحبه حدود 30 دقیقه زمان شما را خواهد گرفت. اگر شما موافقت کردید که در این مطالعه شرکت کنید، از شما سوالاتی راجع به دانش شخصی تان در مورد افرادی که رفتارهای پرخطر دارند در این هات سپات و همچنین در هات سپات ها دیگر شهر یا منطقه خواهیم پرسید. برخی از نمونه سوالاتی که ما خواهیم پرسید این است:

- موقعیت هات سپات ها که در ان اکثرآ جمعیت کلیدی یکجا میشوند
- تخمین تعداد افرادی که رفتار های پرخطر دارند و مکررآ از این هات سپات ها بازدید میکنند.
- موجودیت مراکز و دست رسی به خدمات صحی و دیگر خدمات که برای جمعیت های کلیدی خدمات عرضه میکنند مثلا (sober houses )

ما در جریان مصاحبه نوت میگیریم. این نوت همرای ما کومک میکند که ما ریکارد دقیق برای نوشته کردن راپور ما از موضوعات که در جریان مصاحبه گفته میشود داشته باشیم .

**فواید.**

شما ممکن است به طور مستقیم از مطالعه بهره مند نشوید؛ شما یا کسی که شما میشناسید ممکن است از این مطالعه به طور غیرمستقیم سود ببرد، زیرا آنچه که ما یاد میگیریم، به ما کمک میکند تا خدمات را بهبود بخشد و دست رسی به افرادی که ضرورت دارند بیشتر شود.

**خطرات یا ناراحتی**

یک خطر جزئی وجود دارد که شما می توانید با نقش و نوع کاری که انجام می دهید شناسایی شوید. این کاملا قطعی نیست که شما شناسایی نمیشوید. ما از نام شما استفاده نمی کنیم و کوشش زیادی خواهیم کرد تا از حریم خصوصی شما محافظت کنیم.

**محرمانه بودن**

ما ااسم و یا سایر اطلاعات شناسایی شما را ضبط نخواهیم کرد. ما مصاحبه شما را با یک عدد کود گذاری می کنیم تا به ما در ردیابی اطلاعات مان از مصاحبه کمک کند. هیچ ارتباطی با نام شما در هر زمان وجود نخواهد داشت. ما این نوت ها را بعد از مطالعه از بین می بریم.

کارکنان تمامی این مطالعه توافق نامه نگهداشت محرمیت را امضا کرده اند. کارکنان مطالعه با کارفرمای شما و یا هر کسی که خارج از تیم تحقیق است، در مورد آنچه که آنها یاد می گیرند یا در جریان مصاحبه میشنوند صحبت نمی کنند.

**هزینه / پرداخت**

برای اشتراک در مطالعه هزینه ای وجود ندارد.

**جبران خسارت**

شرکت شما داوطلبانه است. ما هیچ هدیه مالی و غیر مالی به شما نمی دیهیم.

**حق انتخاب با شما است**

شما می توانید انتخاب کنید که در مطالعه شرکت نخواهید کرد. اگر در مطالعه شرکت نمی کنید، مجازات نیست. اگر تصمیم گرفتید که در این مطالعه شرکت کنید، می توانید در هر زمان بدون مجازات توقف کنید. شما مجبور نیستید دلیل برای توقف داشته باشید

**افرادی که با انها تماس بیگرید.**

اگر در مورد این مطالعه سوالی دارید، یا باور دارید که به علت اشتراک در مطالعه برای شما کدام آسیب رسیده است، میتوانید با :

داکتر نقیب الله همدرد، ریس پروگرام ملی کنترول ایدز و هیپاتیت وزارت صحت عامه.

نمبر تلیفون: ۰۷۹۵۵۹۰۷۷۲ تماس بگیرید.

اگرشما در مورد حقوق شما به حیث اشتراک کننده سوال دارید یا میخواهید که نقض را گذرش دهد لطفا با:

داکتر عبدالرشید مسول پروژه در افغانستان وریس موسسه انکشافی و صحی برای جوانان

نمبر تلیفون: ۰۷۰۰۰۷۲۱۰۹ تماس بگیرید.

آیا در مورد آنچه که من گفتم، سؤالی دارید؟

آیاموافق هستید که در مصاحبه شرکت کنید؟
