# Supplementary material for: Mapping and population size estimates of people who inject drugs in Afghanistan in 2019: Synthesis of multiple methods
Source: PLoS One. 2022 Jan 28;17(1):e0262405. doi: 10.1371/journal.pone.0262405 (PMC8797259; doi:10.1371/journal.pone.0262405)
Supplement: S2 Appendix — (ZIP) [file pone.0262405.s002.zip › PWID-Dari Tools/Appendix 15. Consent Form for Focus Group Discussion.docx]

# ضمیمه ۱۵: فورم رضایت نامه برای بحث ګروهی

موسسه صحی و انکشافی برای جوانان( YHDO) از شما دعوت میکند که در تحقیق برآورد جمعیت و نقشه برداری سه جمعیت کلیدی در افغانستان در سال 2019 شرکت کنید. معلومات که من به شما ارائه می دهم می تواند به تصمیم گیری شما در مورد شرکت در مطالعه کمک کند.

1. **چرا ما این مطالعه را انجام میدهیم.**

افرادی که مواد را تزریق می کنند یا رفتارهای پر خطر جنسی دارد در معرض برخی مشکلات صحی مانند اچ آی وی قرار دارند. در همکاری با وزارت صحت عامه افغانستان، ما ارزیابی مینمایم تا بیشتربدانیم که جمعیت ها در کدام قسمت ها در هشت شهر افغانستان قابل دسترسی است. اطلاعات جمع آوری شده در مورد این مطالعه برای هدفگیری اقدامات پیشگیری با این جمعیت ها مورد استفاده قرار می گیرد.

1. **مراحل مطالعه**

اگر موافق هستید که در این مطالعه یا بحث گروهی باشید، مراحل زیر انجام خواهند شد.

- از شما خواسته خواهد شد که در بحث گروهی با حدود 6 تا 10 نفر دیگر شرکت کنید تا در مورد موقعیت های هات سپات ها در شهر که در ان استفاده مواد مخدر ویافعالیت های جنسی صورت می گیرند بحث کنیم. همچنان تخمین شما در باره تعداد این جمعیت ها.
- در دوران بحث گروهی ما دو نفر از کارکنان مطالعه شرکت می کنیم. یکی از ما نوت /یاداشت موضوعات کی بحث می شود بر روی کاغذ می گیرد.
- ما می خواهیم که بحث به صورت صوتی ثبت کنیم، بنا بر این می توانیم بعدا آنرا گوش دهیم و مطمئن شویم که هیچ نظری از دست نداده ایم. اگرشما نمی خواهید که بحث گروهی به صورت صوتی ثبت شود لطفا به ما خبر بدهد. در این صورت ما فقط نوت میگیریم. بعد از نوت گرفتن ما ثبت از بین میبریم.
- برای حفظ محرمیت شما، ما از شما تقاضا میکنیم که در دوران بحث نام خود را استفاده نه کنید. شما میتوانید که از یک نام مستعاریا نمبر مشخص استفاده کنید .

1. **بحث گروهی در کجا انجام خواهد شد**

بحث گروهی در شهر کابل یا شهر های دیگر افغانستان که برای این سروی انتخاب شده انجام می شود.

1. **تمویل کننده، همکار و تطبیق کننده مطالعه**

این مطالعه توسط برنامه UNDP / Global Fund تمویل شده است. کارشناسان وزارت بهداشت، و دانشگاه کالیفرنیا سان فرانسیسکو و دانشگاه علوم پزشکی کرمان، این مطالعه را طراحی کرده اند وداتا را تجزیه و تحلیل خواهند کرد. YHDO تطبیق، جمع اوری داتا ونظارت بر تطبیق و پیشرفت مطالعه مطابق پروتوکول مطالعه به عهده دارد.

1. **تعداد اشتراک کنندگان**

در کل، حدود 30 نفر در بحث های گروهی در هر شهر شرکت خواهند کرد.

1. **مدت زمان**

بحث گروهی حداکثر 1.5 ساعت طول خواهد کشید.

1. **موارد مورد توجه و خطرات احتمالی**

اشتراک در این مطالعه برخی از خطرات وجود دارد:

- شما ممکن است در صحبت کردن در مورد استفاده مواد مخدر و فعالیت های جنسی احساس ناراحتی کنید.
- از شما و تمامی شرکت کنند گان بحث گروهی خواسته می شود که آنچه در بحث گروهی بحث شد محفوظ نگهدارید. اما احتمال دارد که شرکت کنندگان بحث گروهی به دیگر افراد بیرون بحث گروهی سخن بگویند.

1. **محرمیت**

ما هرکار را میکنیم تا محرمیت شما را نگهداریم. انجام دهیم. اسم و یا سایر اطلاعات شناسایی شما را خواسته نمی شود. ما مصاحبه شما را با یک عدد کود گذاری می کنیم تا به ما در ردیابی اطلاعات مان از مصاحبه کمک کند. ما از شما خواهش میکنیم که در طول گروه بحث خود از نام کامل خود استفاده نکنید و ما هیچ یک از اطلاعات شخصی شما را نخواهیم پرسید.

1. **فواید**

شما ممکن است به طور مستقیم از مطالعه بهره مند نشوید؛ شما یا کسی که شما میشناسید ممکن است از این مطالعه به طور غیرمستقیم سود ببرد، زیرا آنچه که ما یاد میگیریم، به ما کمک میکند تا خدمات را بهبود بخشد و دست رسی به افرادی که ضرورت دارند بیشتر شود

1. **حق انتخاب**

شما ازاد هستی انتخاب کنید که در مطالعه شرکت نخواهید کرد. اگر در مطالعه شرکت نمی کنید، مجازات نیست. این مطالعه کاملا داوطلبانه است.

1. **جبران خسارت**

به شرکت کنندگان بحث گروهی غذا و هزینه سفر داده می شوند.

1. **افرادی که با انها تماس بیگرید.**

اگر در مورد این مطالعه سوالی دارید، یا باور دارید که به علت اشتراک در مطالعه برای شما کدام آسیب رسیده است، میتوانید با :

داکتر نقیب الله همدرد، ریس پروگرام ملی کنترول ایدز و هیپاتیت وزارت صحت عامه.

نمبر تلیفون: ۰۷۹۵۵۹۰۷۷۲ تماس بگیرید.

اگرشما در مورد حقوق شما به حیث اشتراک کننده سوال دارید یا میخواهید که نقض را گذرش دهد لطفا با:

داکتر عبدالرشید مسول پروژه در افغانستان وریس موسسه انکشافی و صحی برای جوانان

نمبر تلیفون: ۰۷۰۰۰۷۲۱۰۹ تماس بگیرید.

1. **هزینه/پرداخت**

برای اشتراک در مطالعه هزینه ای وجود ندارد.

1. **توافق**

آیا در مورد آنچه که من گفتم، سؤالی دارید؟

شرکت در مطالعه یا بحث گروهی داوطلبانه است. اگر شما نمی خواهید که شرکت کنید، شما ازاد هستی که بحث گروهی را ترک کنی. اگر می خواهید شرکت کنید، لطفا اینجا بمانید و ما بحث گروهی را انجام می دهیم.
