# Supplementary material for: Mapping and population size estimates of people who inject drugs in Afghanistan in 2019: Synthesis of multiple methods
Source: PLoS One. 2022 Jan 28;17(1):e0262405. doi: 10.1371/journal.pone.0262405 (PMC8797259; doi:10.1371/journal.pone.0262405)
Supplement: S2 Appendix — (ZIP) [file pone.0262405.s002.zip › PWID-Dari Tools/Appendix 22- Supervision form-Dari Language.docx]

# ضمیمه ۲۲: فورم نظارت

سوپروایزر شهری این فورم نظارت را هر وقت که با تیم سروی در جلسات صبح در دفتر یا در هات سپان دیدار می کند، تکمیل می کند.

جمعیت هدف: PWID WHRB MHRB

اگر این فرم برای هات سپات خاصی تکمیل شده باشد، شناسه ها و آدرس ها را بنویسید:

تاریخ: ...../....../....... کود تیم سروی: ........... شهر:.........................

| اگر این فورم برای یک هات سپات مشخص تکمیل شده باشد، ID ان هات سپات و ادرس ان نوشته کنید: |
| --- |
| ……………………………………………………………………..…………………………………………………..…..…. هات سپات : ID  …………………..…………………………………………………………………………..………………………… ادرس هات سپات : |

1. ارزیابی سر تیم

| بلی نخیر ارزیابی نشد | 1. تیم هات سپات را به تاریخ /وقت پلان شده بازدید کرد؟ |
| --- | --- |
| بلی نخیر ارزیابی نشد | 1. اقدامات امنیتی با دقت مورد ارزیابی و مدیریت شد؟ |
| بلی نخیر ارزیابی نشد | 1. هر گونه خشونت یا واقعات گزارش یا مشاهده شده است؟ |
| بلی نخیر ارزیابی نشد | 1. فورم شمارش هیچ داتا کلیدی را از دست نداده؟ |
| بلی نخیر ارزیابی نشد | 1. GPS کود به درستی ثبت شده است؟ |
| بلی نخیر ارزیابی نشد | 1. تعداد مصاحبه ها با اعضای کلیدی جمعیت 2 تا 8 بود؟ |
| بلی نخیر ارزیابی نشد | 1. تعداد مصاحبه ها با دیگر معلومات دهنده کلیدی 3 تا 5 بود؟ |
| بلی نخیر ارزیابی نشد | 1. مدت زمان حضور در هات سپات ها حد اقل 2 ساعت است؟ |

اگر پاسخ به هر یک از سوالات فوق نخیر باشد، دلایل و علت را بررسی کنید؟ زیر دلایل و اقداماتی راکه برای حل کردن مشکل انجام دادید بنویسید:

………………………………………………………………………………………………………………………………………………………………………………………………………………………………………………………………………………………………………………………………………………………………………………………………………………………………………………………………………………………………………………………………………………………………………………………………………………………………………………………………………………………………………..

2. ارزیابی مصاحبه کننده

| بلی نخیر ارزیابی نشد | 1. سکریننگ برای واجد شرایط به درستی انجام شده بود |
| --- | --- |
| بلی نخیر ارزیابی نشد | 1. تنها افراد واجد شرایط ثبت شده اند |
| بلی نخیر ارزیابی نشد | 1. میزان باسخ (فیصدی افراد واجد شرایط که مصاحبه شده بود) بیش از 80% بود |
| بلی نخیر ارزیابی نشد | 1. جمع اوری رضایت نامه به درستی انجام شده بود |
| بلی نخیر ارزیابی نشد | 1. ? یک جای خصوصی برای مصاحبه استعمال شده بود |
| بلی نخیر ارزیابی نشد | 1. داتا از دست رفته برای سوالهای کلیدی صفر است |
| بلی نخیر ارزیابی نشد | 1. توافق بین داتا از بخش ها و سوالات مختلف |
| بلی نخیر ارزیابی نشد | 1. مشوق در پایان مصاحبه به طور مناسب داده شده |

اگر پاسخ به هر یک از سوالات فوق نخیر باشد، دلایل و علت را بررسی کنید؟ زیر دلایل و اقداماتی راکه برای حل کردن مشکل انجام دادید بنویسید:

…………………………………………………………………………………………………………………………………………………………………………………………………………………………………………………………………………………………………………………………………………………………………………………………………………………………………………………………………………………………………………………………………………………………………………………………………………………………………………………………………………………………………………
