# Supplementary material for: Mapping and population size estimates of people who inject drugs in Afghanistan in 2019: Synthesis of multiple methods
Source: PLoS One. 2022 Jan 28;17(1):e0262405. doi: 10.1371/journal.pone.0262405 (PMC8797259; doi:10.1371/journal.pone.0262405)
Supplement: S2 Appendix — (ZIP) [file pone.0262405.s002.zip › PWID-Dari Tools/Appendix 6. Venue Observation and Enumeration Form.docx]

**ضمیمه۶: فورمه مشاهده هات سپات و شمارش**

| اسم سرتیم...................................  اسم سرویر......................................... | شهر:.........................  نمبرهات سپات:..............................  ادرس هات سپات:(ناحیه ،گذریا قریه،سرک،کوچه)........................... |
| --- | --- |
| تاریخ تکمیل فورم (روز/ ماه / سال)........................... |  |
| زمان ورود به هات سپات(۲۴ ساعته:ساعت:دقیقه) ................  ختم هات سپات(۲۴ ساعته:ساعت: دقیقه) ........................  جمعیت مورد هدف.............................................  ☐ PWID ☐ MHRB ☐ WHRB  دفعه بازدید از هات سپات☐نوبت اول ☐نوبت دوم |  |
|  |  |
|  |  |
|  |  |

**کواردینات GPS ساحه**

| (طول البلد)Longitude ______________ | (عرض البلد)Latitude ______________ |
| --- | --- |
| ______________ (موبایل کود) |  |

**نوعیت هات سپات:**

☐خانه های ترک شده/ویران شده ☐پارک ☐مکان های کوچه ☐رستوارنت /قهوه خانه

☐فروشگاه ☐استادگاه موترها ☐هوتل/سرای ☐خانه شخصی
☐دیگر (ذیل توضیح کنید)

توضیح مختصر هات سپات:

.........................................................................................................................................................................................................................................................................

**تعداد افراد توسط شمارش مستقیم**

| جمعیت | مجموع | به اساس جنسیت | | به اساس عمر | |
| --- | --- | --- | --- | --- | --- |
|  |  | تعداد مردها | تعداد زن ها | تعداد افراد که عمر شان از ۲۵ سال کم باشد | تعداد افراد که عمر شان از ۲۵ سال زیاد باشد |
| PWID |  |  |  |  |  |
| MHRB |  |  |  |  |  |
| WHRB |  |  |  |  |  |

NA: Not Applicable (در هر خانه کی قابل تطبیق نه باشد باید NAنوشته شود )

**تعداد افراد که برای سروی مختصر رسیدگی شده**

| **تعداد رد از اشتراک** | **تعداد رسید گی** | **جمعیت** |
| --- | --- | --- |
|  |  | PWID |
|  |  | MHRB |
|  |  | WHRB |

فعالیت های مشاهده شده در هات سپات

☐**به دنبال شریک جنسی/مشتری ☐فروشنده گان مواد مخدر☐استعمال مواد مخدر ☐تزریق مواد مخدر**

**☐داشتن رابطه جنسی ☐محل برای زندگی ☐دیدن دوستان ☐دیگر ـتوضیح بدهد**

**توضیح مختصر نوعی دیگری از فعالیت های در هات سپات**

**_____________________________________________________________________
_____________________________________________________________________________________**

علایم مشاهده شده در هات سپات

☐سیرنج/سوزن ☐دیگر وسایل استفاده مواد مخدر ☐کاندوم ☐دی**گ**ر (مشخص نماید)ـــــــــــــــــــــــــــــ---

ایا شما قادر به عکس گرفتن از هات سپات بودید؟ ☐بلی ☐نخیر

ایا این مشاهده زمانی بود که ساحه بشترین جمعیت مورد هدف شده داشت **؟**

☐نخیر ☐بلی

**دیگر تبصره یا دیگر موضوعات در اثنایی مشاهده عملی از هات سپات**

**______________________________________________________________________
_____________________________________________________________________**

**_____________________________________________________________________________________
_____________________________________________________________________________________**

**_____________________________________________________________________________________**
