# Supplementary material for: Mapping and population size estimates of people who inject drugs in Afghanistan in 2019: Synthesis of multiple methods
Source: PLoS One. 2022 Jan 28;17(1):e0262405. doi: 10.1371/journal.pone.0262405 (PMC8797259; doi:10.1371/journal.pone.0262405)
Supplement: S2 Appendix — (ZIP) [file pone.0262405.s002.zip › PWID-Dari Tools/Appendix 7. PWID Screening and Data Collection Form.docx]

# ضمیمه ۷: سکریننگPWID و فورم جمع اوری اطلاعات

ما یک مطالعه را انجام میدهیم برای اینکه بهتر بفهمیم که در کجاها میتوانیم اشخاصی را پیدا نمایم که خدمات پیشگیری برای شان انجام بدهیم. برای اینکه شما در این مطالعه سهم بیگیرد از همه اولتر ما چند سوالها را از شما خواهیم پرسید. جوابات شما ما را کمک میکند که شما واجد شرایط هستین که در این مطالعه شرکت نماید یا خیر. اگر شما واجد شرایط بودید ما فورم موافقیت را با شما تکرار میکنیم. تمامی جوابات شما نزد ما مخفوظ میباشد به این معنی که با هیچ کسی شریک نمی سازیم. اگر شما واجد شرایط بودید ما ازشما خواهیم خواست که در این مطالعه شرکت نماید. این مطالعه کمتر از ده دقیقه وقت شما را خواهد گرفت و برای شما ۷۵ پرداخت خواهد شد مطابق وقتی که شما با ما گذرانید.

( سوالات شرایط شایستگی)

| **جواب** | **سوال** |
| --- | --- |
| پشتو  دری  ازبکی  دیگر (سکریننګ متوقف نماید. فرد واجد شرایط نیست) | ۱.در کدام زبان ها شما تسلط دارید؟ (حداقل در یکی از زبان ها تلسط داشته باشد تا برای این مطالعه واجد شرایط شمرده شود). |
| --------- سال ( اگر۱۷سال یا پایین تر عمر داشت. واجد شرایط نیست) | ۲. چند سال دارید؟ |
| **بلی ( به سوال ۴ ادامه بدهید)**  **نخیر( سکریننگ را توقف کنید، شخص واجد شرایط نیست)** | ۳.ایا شما در ۱۲ ماه گذشته کدام مواد مخدر استفاده کرده اید؟ |
| **بلی ( به سوال ۵ ادامه بدهید)**  **نخیر( سکریننگ را توقف کنید، شخص واجد شرایط نیست)** | ۴.ایا شما در ۱۲ ماه گذشته کدام مواد مخدر تزریق کرده اید؟ |
| **بلی (سکریننگ را توقف کنید، شخص واجد شرایط نیست)**  **نخیر( به سوالهای دیموگرافیک و مواد مخدر ادامه بدهد)** | ۵. آیا شما در همچنین مطالعه در سه ماه گذشته اشتراک کرده اید؟ |

**[اگر شخص واجد شرایط باشد رضایت شفاهی را دریافت کند]**

**سوالات مربوط به دیموگرافیک و مواد مخدر**

| **کود** | **جواب** | **سوال** | **Q#** |
| --- | --- | --- | --- |
| 1  2  3 | مرد  زن  دیگر (مشخص نماید)_______________ | جنسیت | ۱ |
| 1  2  3 | در یک ماه گذشته  در سه ماه گذشته  در ۱۲ماه گذشته | اخرین باری که مواد تزریق کردی چی زمانی بود؟ | ۲ |
| 1  2  3  4  5  6 | هیروین  کوکایین  تریاک  امفی تامین  دواهای نسخه یی  دیگر مشخص کنید_____________________ | **کدام مواد مخدررا شما بیشتر تزریق میکنید ؟**  نوت: **هرچند که قابل تطبیق باشد نشانی کنید.** | ۳ |

پرسش ها برای برآورد اندازه جمعیت

| **کود** | **جواب** | **سوال** | **Q#** |
| --- | --- | --- | --- |
| 1  2  3 | بلی  نخیر  مطمین نیستم | ایا شما در مطالعه دیگری درسال ۲۰۱۲ شرکت کردید که سوالات مثلا معاینه خون برای HIV ازشما پرسیده میشد وبرای شما کوپون داده میشد تا دوست تان را هم برای اشتراک در مطالعه پیدا کنید؟ | ۴ |
| 1  2 | بلی  نخیر | ایا شما در گذشته کلاه زمستانی شبیه به این کلاه زمستانی از افرادی که آنها را در هات سپات ها توزیع میکردند دریافت کرده اید؟ | ۵ |
| 1  2 | بلی  نخیر | ایا شما در ۱۲ماهگذشته خدمات ... از مرکز...بدستاورده اید؟ | ۶ |
| 1  2 | بلی  نخیر | ایا شما در ۱۲ماه گذشته خدمات ... از مرکز...بدستاورده اید؟ | ۷ |
| ---- | *مجموعه :حداقل ....................حداکثر----------------*  *مردان : حداقل.......................حداکثر----------------*  *زنان : حداقل ........................حداکثر----------------* | بنظر شما چند نفر دیگر که مواد مخدر تزریق میکنند دراین شهر ............ زندگی میکند.  نوت: اگر تعداد بسیارکم یا زیاد باشد (مثلا اضافه تراز جمعیت این شهر) از شخص بخواهید که دوباره فکر نماید. همچنان تعداد زنان و مردان مساوی به تعداد کل شود. | ۸ |

سوالات درباره این هات سپات

| **Code** | **جواب** | **سوال** | **Q#** |
| --- | --- | --- | --- |
| 1  2  3  4  5  6  7  8 | 1 – 5 مرتبه  6 – 10 مرتبه  11 – 15 مرتبه  16 – 20 مرتبه  21 – 25 مرتبه  26+ مرتبه  نمی دانم  ازجواب دادن انکار کرد | در ۳۰ روزی گذشته شما چند بار از این محل دیدن کردید ؟ | ۹ |
|  | روز............................................................  وقت.............................................................  تاریخ مشخص (روز/ماه/سال)................................. | چی زمانی این هات سپات بیشترین جمعیت استفاده کنندگان ویا تزریق کنندگان مواد مخدر دارد ؟ | ۱۰ |
|  | اقایان......................................................  خانم ها ..................................................... | به چی تعداد اشخاص مختلف را شما در هفت روز گذشته در این هات سپات دیدید که مواد مخدر تزریق میکند؟ | ۱۱ |
|  | اقایان...........................................................  خانم ها.......................................................... | به چی تعداد اشخاص مختلف را شما در ۳۰ روز گذشته در این هات سپات دیدید که مواد مخدر تزریق میکند؟ | ۱۲ |
| 1  2  3  4  5  6 | سوزن یا سرنج رایگان  کندم رایگان  مشاوره و معاینه اچ آی وي رایگان  مواد تعلیمی وقایوی رایگان  دیگر لطفا ذکرکنید ________________)  هیچ تا حدیکه ما می دانم | کدام یک از خدمات زیر در این هات سپات در طول ماه گذشته ارائه شده است؟ | ۱۳ |
| 1  2  3 | بلی  نخیر  نمی دانم | آیا پلیس در ماه گذشته از این هات سپات بازدید یا گشت و گذار کرده است؟ | ۱۴ |

سوال در مورد سه هات سپات دیگر که در آن فرد اکثر بازدید می کند

| **Code** | **جواب** | **سوال** | **Q#** |
| --- | --- | --- | --- |
| 1  2  3  4  5 | من به محلات دیگر نمی روم (**بروند Q22به سوال** )  یک محل  دو محل  سه محل  از سه محل اضافه(چند ________) | شما به چند هات سپات دیگراکثرا برای تزریق مواد مخدر یا ملاقات با دیگران که مواد مخدر تزریق می کند، مراجعه می کنید؟ | ۱۵ |
|  | نام ....................................  ادرس....................................................... | نام / آدرس هات سپات دیگریکه اغلب شما به آنجا میروی برای تزریق مواد مخدر یا ملاقات با دیگرانی که مواد مخدر تزریق میکنند؟ | ۱۶ |
| 1  2  3  4  5  6  7  8  9 | 1 – 5 مرتبه  6 – 10 مرتبه  11 – 15 مرتبه  16 – 20 مرتبه  21 – 25 مرتبه  26+ مرتبه  نمی دانم  ازجواب دادن انکار کرد  قابل تطبیق نیست | در 30 روز گذشته، چند بار شما از این هات سپات بازدید کردید؟ | ۱۷ |
|  | نام...........................................  ادرس......................................... | نام / آدرس هات سپات دوم که اغلب شما به آنجا میروی برای تزریق مواد مخدر یا ملاقات بادیگرانیکه مواد مخدر تزریق میکنند؟ | ۱۸ |
| 1  2  3  4  5  6  7  8  9 | 1 – 5 مرتبه  6 – 10 مرتبه  11 – 15 مرتبه  16 – 20 مرتبه  21 – 25 مرتبه  26+ مرتبه  نمی دانم  ازجواب دادن انکار کرد  قابل تطبیق نیست | در 30 روز گذشته، چند بار شما از این هات سپات بازدید کردید؟ | ۱۹ |
|  | نام.................................................  ادرس............................................. | نام / آدرس هات سپات سوم که اغلب شما به آنجا میروی برای تزریق مواد مخدر یا ملاقات با دیگرانی که مواد مخدر تزریقمیکنند؟ | ۲۰ |
| 1  2  3  4  5  6  7  8  9 | 1 – 5 مرتبه  6 – 10 مرتبه  11 – 15 مرتبه  16 – 20 مرتبه  21 – 25 مرتبه  26+ مرتبه  نمی دانم  ازجواب دادن انکار کرد  قابل تطبیق نیست | در 30 روز گذشته، چند بار شما از این هات سپات بازدید کردید؟ | ۲۱ |

**سوالات رفتاری**

| 1  2  3  4  5  6 | مجرد  متاهل و با همسرم زندگی میکنم  متاهل اما با همسرم زندگی نمیکنم  ازدواج نکردم اما با رفیقم یا شریکم زندگی میکنم  طلاق شده/جدا شده  بیوه | وضعیت تاهل فعلی شما چیست؟ | ۲۲ |
| --- | --- | --- | --- |
| 1  2 | بلی  نخیر (به سوال ۲۷ بروید) | **(برای مردان**) آیا تا به حال رابطه جنسی مقعدی یا دهانی با یک مرد دیگر داشته اید؟ | ۲۳ |
| 1  2 | بلی  نخیر | **(برای مردان**) آیا در 12 ماه گذشته رابطه جنسی مقعدی یا دهانیبا یک مرد دیگر داشته اید؟ | ۲۴ |
| 1  2  3 | بلی  نخیر, من درمقابل پول ومواد مخدر رابطه جنسی داشتیم، اما در ۱۲ ماه اخر نخیر  نخیر, من هرگز در بدل پول و مواد مخدر رابطه جنسی نداشته ایم | ***(برای مردان)*** *آیا شما در 12 ماه گذشته در مقابل پول یا مواد مخدر فعل جنسی مقعدی یا دهانیرا تبادله کرده اید؟*  **اگر نه، آیا برای دایم نخیر یا صرف در 12 ماه گذشته ؟** | ۲۵ |
| 1  2  3 | بلی  نخیر, من درمقابل پول ومواد مخدر رابطه جنسی داشتم، اما در ۳ ماه اخر نخیر  نخیر، من هرگز در بدل پول و مواد مخدر رابطه جنسی نداشته ایم | ***(برای خانم ها)*** *آیا در 3 ماه گذشته، در مقابل پول یا مواد مخدر رابطه جنسی مهبلی، مقعدی یا دهانی داشتی؟*  **اگر نه، آیا برای دایم نخیر یا صرف در ۳ ماه گذشته ؟** | ۲۶ |
| 1  2 | بلی  نخیر**( به بخش دیگربروید)** | شده اید ؟HIVآیا تا به حال ازمایش | ۲۷ |
| 1  2  3 | درحدود ۱۲ ماه گذشته  1-2 سال قبل  اضافه تر از ۲ سال | چی وقت بوده؟HIV اخرین ازمایش | ۲۸ |
| 1  2 | بلی  نخیر **(به بخش دیگر بروید)** | آیا حالت اچ آی وی خود را می فهمی؟ | ۲۹ |
| 1  2  3 | HIV منفی  HIV مثبت  راحت احساس نمی کند که اظهار کند | اگر شما راحت هستین میشود بگوید، حالت شما چیست؟ | ۳۰ |

بخش اختتام

|  | نمبر هات سپات |
| --- | --- |
|  | نمبر اشتراک کتنده |
|  | نام مصاحبه کننده |
|  | تاریخ تکمیل فورم (روز/ماه/سال) |
