# Supplementary material for: Mapping and population size estimates of people who inject drugs in Afghanistan in 2019: Synthesis of multiple methods
Source: PLoS One. 2022 Jan 28;17(1):e0262405. doi: 10.1371/journal.pone.0262405 (PMC8797259; doi:10.1371/journal.pone.0262405)
Supplement: S3 Appendix — (ZIP) [file pone.0262405.s003.zip › PWID-Pashto Tools/Appendix 3--Key Informant Interview and FGD guide for PWID.docx]

# ضمیمه ۳: رهنمود مصاحبه انفرادی شخص کلیدی وبحث گروپی برایPWID

**مکان شناسی استفاده کنندگان زرقی مواد مخدر**

**نمبر مصاحبه:........................................**

**نام مصاحبه کننده/تسهیل کننده بحث گروپی:.................. ................**

**نمبر نوت گیرنده: .................................**

**نام نوت گیرنده: .................................**

**تاریخ مصاحبه/بحث گروپی (روز،ماه،سال)...............................................................**

**نوت: ( قبل از بحث گروهی رضایت نامه شفاهی از اشتراک کنندگان باید گرفته باشید. همچنان برای مصاحبه شخص کلیدی و کارکنان ارگان های غیر دولتی/دولتی که خدمات عرضه میکنند باید رضایت نامه شفاهی داشته باشید)**

**مقدمه**

تشکر از اینکه موافقت کردید که در این مصاحبه شرکت کنید. به یاد داشته باشید که تمام جوابات شما محرمانه نگه داشته می شود ومی توانید مصاحبه را در هر زمان متوقف نماید. شما می توانید که مصاحبه را هر زمانی که خواسته باشید متوقف نماید. لطفآ نام شخص خودی تان یا اشنایی تان را ذکر نه نماید. شما میتواند مخفف یا کدام نام مستعار استفاده کنید.

**از تمام شرکت کنندگان پرسیده شود**.

۱. لطفا برایم درباره نواحی شهر......................(نام شهر) که در آنفعالیتمواد مخدر صورت میگیرد بگوید؟ فعالیت های مواد مخدر شامل فروش، استفاده و تزریقمیشوند. اگر مشخصآ ذکر نکرد شما درباره استفاده زرقی مواد مخدر سوال کنید.

**(نقشه کاغذی یا دیجیتلی که دارای مرزهای روشن نواحی باشد برای بحث تان استفاده کنید)**

۲. اکنون کمک شما را میخواهم در ایجاد یک لست هات سپات ها به اساس نواحی شهر. (۷ ستون جدول را ایجاد کنید ستون ۱ ناحیه شهری

1. در این نواحی مختلف کدام قسم هات سپات ها هستند؟ [ در ستون دوم موقعیت/نوع داخل کنید. این کمک میکند که ما انواع مکان ها و فاصله های فضایی انها پیدا کنیم. مثلا عقب بازار مرکزی/ مارکیت خرابه)
2. لطفا به من بگویید که چند نفر از مصرف کنندگان مواد مخدر (از جمله کسانی که مواد مخدر را نیز تزریق می کنند) - مرد و زن - در هر یک از این هات سپات های که ذکر کرده اید، در روز معمولی جمع میشوند؟ [داخل ستون 3]
3. لطفا به من بگویید که چند نفر از تزریق کنند گان مواد مخدر - مرد و زن - در هر یک از این هات سپات های که ذکر کرده اید، در روز معمولی جمع میشوند؟ [داخل ستون ۴]
4. لطفا به من بگویید که چند نفر از تزریق کنند گان هیروین - مرد و زن - در هر یک از این هات سپات های که ذکر کرده اید، در روز معمولی جمع میشوند؟ و چند نفر از این ها زنان هستند؟ [داخل ستون ۵]
5. لطفا روز و وقت را به من بگویید که مصرف کنند گان مواد مخدر/ تزریق کنندگان از این هات سپات بازدید میکند [داخل ستون ۶)
6. لطفا روز و وقت به من بگویید که بیشترین تعداد مصرف کنندگان مواد مخدر/ تزریق کنندگان از این هات سپات بازدید میکند؟ (داخل ستون ۷)

| **7** | **6** | **5** | | **4** | | **3** | | **2** | **1** |
| --- | --- | --- | --- | --- | --- | --- | --- | --- | --- |
| اوج روز/وقت | تمام روز ها/وقت | تعداد تزریق کننده گان هروین | | تعداد تزریق کننده گان مواد مخدر | | تعداد مجموعی استفاده کنند گان | | ادرس هات سپات/ نوع هات سپات | ناحیه شهری |
|  |  | M | F | M | F | M | F |  |  |
|  |  |  |  |  |  |  |  |  |  |
|  |  |  |  |  |  |  |  |  |  |
|  |  |  |  |  |  |  |  |  |  |
|  |  |  |  |  |  |  |  |  |  |
|  |  |  |  |  |  |  |  |  |  |

**و ارایه کننده گان خد مات سوال نمایدNGOs از**

۳. لطفا رابطه و تعامل موسسه تان را با مصرف کنده گان تزریق کنندگان مواد مخدر توضیح بدهد-چی نوع خدمات را برای انها ارایه میکنید. در صورتیکه وجود داشته باشد؟

**۴**.چی تعداد مصرف کنده گانیا تزریق کنده گانمواد مخدر دریک ماه به خدمات شما دسترسی دارد؟**۵**. چگونه به مصرف کننده گان مواد مخدر دسترسی پیدا می کنید؟

**۶**. چه فکر میکند راجع به موانع در مقابل دسترسی مصرف کننده گان یا تزریق کننده گان مواد مخدر به خدمات صحی چی میباشد؟

**۷**. ایا شما از دیگر کدام موسسات که خدمات صحی ویا دیگرخدمات برای مصرف کننده گان یا تزریق کنده گان مواد مخدر ارایه میکند، اگاه هستید؟

**۸**. بنظر شما مصرف کننده گان مواد مخدر بیشتر به کدام نوع خدمات صحی نیاز دارد؟.

**بحیث معلومات دهنده یی کلیدی بپرسید. PWIDاز**

**۳**.از کدام نوع خدمات صحی برای مصرف کننده گان مواد مخدر شما اگاهی دارید؟ ازمایش اچ ای وی ، ازمایش هپاتییت/واکسین، متادون، تبادله سوزن، تداوی اعتیاد، خدمات حقوقی.

**۴**.ایا شما از این نوع خدمات استفاده میکنید؟ کدام خدمت؟ چند وقت؟ ایا شما قادر به گرفتن سوزن پاک هستین زمانیکه نیاز داشته باشی؟ اگر نخیر، چرا؟ اگر بلی، در کجا؟

**۵**.بنظر شما کدام نوع خدمات کمبود یا نیاز است؟

**۶**. چی فکر میکنید، مشکلات اساسی شما برای دسترسی به خدمات صحی چی میباشد؟

**۷**. چگونه با مصرف کننده گان مواد مخدر در شهر برخورد میشود؟ با تزریق کننده گان مواد مخدر چگونه رفتار میشود در شهر؟
