# Supplementary material for: Mapping and population size estimates of people who inject drugs in Afghanistan in 2019: Synthesis of multiple methods
Source: PLoS One. 2022 Jan 28;17(1):e0262405. doi: 10.1371/journal.pone.0262405 (PMC8797259; doi:10.1371/journal.pone.0262405)
Supplement: S3 Appendix — (ZIP) [file pone.0262405.s003.zip › PWID-Pashto Tools/Appendix 6. Venue Observation and Enumeration Form.docx]

**ضمیمه۶: د هاټ سپاټ څخه دلیدنې او شمارلو فورمه**

| د سر ټیم نوم...................................  د سرویر نوم......................................... | ښار:.........................  د هاټ سپاټ کود یا نمبر:............................  د هاټ سپاټ ادرس:(ناحیه ،گذریا قریه، سرک، کوڅه) ........................... |
| --- | --- |
| دفورمې د تکمیل نیټه: (ورځ/ میاشت/ کال)........................... |  |
| هاټ سپاټ ته د ننوتلو وخت (۲۴ ساعته:دقیقه:ساعت) ................  د هاټ سپات د پای وخت (۲۴ ساعته:دقیقه: ساعت) ....................  د هدف ګروپ:  ☐ PWID ☐ MHRB ☐ WHRB  د هاټ سپاټ د لیدنې نوبت: ☐ اول نوبت ☐ دوهم نوبت |  |
|  |  |
|  |  |
|  |  |

**د هاټ سپات GPS کواردینات**

| (طول البلد)Longitude ______________ | (عرض البلد)Latitude ______________ |
| --- | --- |
|  |  |

**دهاټ سپاټ ډول:**

☐خالی/خرابه کورونه ☐پارک ☐کوڅه ☐رستوارنټ/قهوه خانه

☐ پلورنځی ☐ د موټرو هډه/استادګاه ☐هوتل/سرای ☐ شخصی کور
☐ نور(لاندی یی واضح کړئ)

دهاټ سپاټ لنډ معلومات:

.........................................................................................................................................................................................................................................................................

**د مستقیم شمارلو په واسطه د افرادو تعداد**

| د هدف ګروپ | مجموع | د جنس په اساس | | د عمر په اساس | |  |
| --- | --- | --- | --- | --- | --- | --- |
|  |  | د نرانو تعداد | د ښځو تعداد | د هغه کسانو تعداد چه عمر یی تر ۲۵ کالو کم وی | د هغه کسانو تعداد چه عمر یی تر ۲۵ کالو زیات وی |  |
| PWID |  |  |  |  |  |  |
| MHRB |  |  |  |  |  |  |
| WHRB |  |  |  |  |  |  |

NA: Not Applicable (باید په هره خانه کی چه قابل د تطبیق نه وی NA ولیکل شی)

**د هغه افرادو تعداد چه د لنډی سروی لپاره ورته رسیدګی وشوه**

| **د ګډون د رد کسانو تعداد** | **د رسیدګی شووکسانو تعداد** | **د هدف ګروپ** |
| --- | --- | --- |
|  |  | PWID |
|  |  | MHRB |
|  |  | WHRB |

په هاټ سپاټ کی لیدل/مشاهده شوی فعالیتونه

☐**دجنسی شریک/مشتری لټول ☐دنشه یی توکو خرڅوونکی ☐د نشه یی توکو استعمال ☐د نشه یی توکو تزریق**

**☐د جنسی اړیکو نیول ☐د ژوند لپاره ځای ☐د ملګروسره لیدل ☐نور (ـتشریح ورکړئ)**

**په هاټ سپاټ کی دنورو فعالیتونو لنډه تشریح توضیح**

**_____________________________________________________________________
_____________________________________________________________________________________**

په هاټ سپاټ کی لیدل شوی علایم

☐سیرنج/ستن ☐د نشه یی توکو د استعمال نور وسایل ☐ کنډم ☐نور (واضح یی کړۍ)ـــــــــــــــــــــــــــــ--

ایا تاسی د هاټ سپاټ څخه عکس واخستو؟ ☐هو ☐نخیر

ایا ستاسی دا لیدنه په داسی وخت کی وه چه په هاټ سپات کی د هدف ګروپ خلک زیات وه؟

☐هو ☐نخیر

**د هاټ سپاټ څخه د لیدنی په دوران کی کومه بله اړوند تبصره یا موضوع**

**______________________________________________________________________
_____________________________________________________________________**

**_____________________________________________________________________________________**
