# Supplementary material for: Mapping and population size estimates of people who inject drugs in Afghanistan in 2019: Synthesis of multiple methods
Source: PLoS One. 2022 Jan 28;17(1):e0262405. doi: 10.1371/journal.pone.0262405 (PMC8797259; doi:10.1371/journal.pone.0262405)
Supplement: S3 Appendix — (ZIP) [file pone.0262405.s003.zip › PWID-Pashto Tools/Appendix 7. PWID Screening and Data Collection Form.docx]

# ضمیمه ۷: د PWID سکریننگ او معلوماتو د راټولو فورمه

مونږ یوه څیړنه کوو تر څو په ښه ډول وپوهیږو چه چیری کولای شو هغه اشخاص پیدا کړو چه نشه یی توکی تزریق کوی ترڅو په راتلونکی کی هغوی ته وقایوی خدمات وړاندی کړو. ددی لپاره چه تاسی په دی څیړنه کی برخه واخلئ، مونږ له تاسی څخه یو څو پوښتنې کوو. ستاسی ځوابونه به له مونږ سره مرسته وکړی چه تاسی د ګډون وړ یاست یا نه. که چیری تاسی د ګډون وړ یاست نو مونږ تاسی د رضایت فورمه تکراروو. ستاسی ټولو ځوابونه به له مونږ سره خوندي پاتی شي. په دی معنی چه له هیڅ یو کس سره یی نه شریکوو. که تاسی د ګډون وړ یاست،نومونږ به له تاسی وغواړو چه په دي څیړنه کی ګډون وکړئ. دا څیړنه شاید د ۱۰ دقیقو څخه کم وخت ونیسي او د هغه وخت په مقابل کی چه تاسی یی له مونږ سره تیروئ، تاسی ته یی په مقابل کی ۷۵ افغانۍ د مرکی په اخر کی درکول کیږي.

( د ګډون د وړتیا پوښتنې)

| **جواب** | **سوال** |
| --- | --- |
| پشتو  دری  ازبکی  نور (**سکریننګ ودروئ. شخص د ګډون وړ نه دی**) | ۱. په کومو ژبو تسلط لرئ؟ ( حد اقل په یو له دی دری ژبو تسلط ولري ترڅو ددی څیړنې لپاره د ګډون وړ وګڼل شي) |
| --------- کال (که چیری ۱۷ کاله یا کم عمر ولري د ګډون وړ نه دی) | ۲. څو کلن یاست؟ |
| **بلی (**۴ سوال ته ادامه ورکړئ**)**  **نخیر(سکریننګ ودروئ. شخص د ګډون وړ نه دی)** | ۳.ایا تاسی په تیرو ۱۲ میاشتو کی کوم نشه یی توکي استعمال کړي دي؟ |
| **بلی (**۵ سوال ته ادامه ورکړئ**)**  **نخیر(سکریننګ ودروئ. شخص د ګډون وړ نه دی)** | ۴. ایا تاسی په تیرو ۱۲ میاشتو کی کوم نشه یی توکي تزریق کړي دي؟ |
| **بلی (سکریننګ ودروئ. شخص د ګډون وړ نه دی)**  **نخیر(** د دیموگرافیک او نشه یی توکو سوالونو ته ادامه ورکړئ**)** | ۵. ایا تاسی په تیرو دری میاشتو کی په دی ډول څیړنه کی ګډون کړی دی؟ |

**[**که شخص د ګډون وړ وي، شفاهی رضایت یی حاصل کړئ]

د دیموګرافیک او نشه یی توکو اړوند سوالونه

| **کود** | **جواب** | **سوال** | **Q#** |
| --- | --- | --- | --- |
| 1  2  3 | نارینه  ښځینه  نور **(مشخص یی کړئ**)_______________ | جنسیت | ۱ |
| 1  2  3 | په تیره ۱ میاشت کی  په تیرو ۳ میاشتو کی  په تیرو ۱۲ میاشتو کی | اخرین ځل مو کله نشه یی توکي تزریق کړل؟ | ۲ |
| 1  2  3  4  5  6 | هیروین  کوکایین  تریاک  امفی تامین  نسخه یی دواګانی  نور (مشخص یی کړئ)_____________________ | **کوم نشه یی توکي تاسی زیات تزریق کوئ؟**  **نوټ:** **هرڅو چه قابل د تطبیق وي په نښه یی کړئ .** | ۳ |

د نفوس د تخمین په هکله پوښتنې

| **کود** | **جواب** | **سوال** | **Q#** |
| --- | --- | --- | --- |
| 1  2  3 | بلی  نخیر  ډاډمند نه یم | ایا تاسی په ۲۰۱۲ کال کی په یوه بله څیړنه کی ګډون کړی وه چه په هغه کی ستاسی څخه د HIV لپاره د وینی د معاینی په هکله پوښتل کیدل. او هم تاسی ته کوپون درکول کیده چه تاسی خپل ملګری په دی څیړنه کی د ګډون لپاره پیدا کړئ؟ | ۴ |
| 1  2 | بلی  نخیر | ایا تاسی په تیره یوه میاشت کی دی خولۍ ته ورته د ژمی خولۍ د هغه کسانوڅخه چه په هاټ سپاټونو کی یی توزیع کولې ترلاسه کړیده؟ | ۵ |
| 1  2 | بلی  نخیر | ایا تاسی په تیرو ۱۲ میاشتو د ADAA مرکز څخه خدمات تر لاسه کړي دي؟ | ۶-a |
| 1  2  3  4  5  6  7  8  9  10 | مشوره دهی(DIC)  اچ آی وی ټسټ  د تور او سپین زیړی ټسټونه (HBS/HCV)  مفت ستن/پاک سیرنح  مفت کنډم  سفلیس تست  تد سفلیس او مقاربتی امراضو تداوي  متاډون تداوي  د تعلیمی او وقایوی موادو توزیع د اګاهی په خاطر  نور مشخص یی کړئ_____________________ | (که ځواب بلی وي)، کوم خدمات تاسی د ADAA مرکزڅخه په لاس راوړي دي؟  (هرڅو چه قابل د تطبیق وي په نښه یی کړئ) | ۶-b |
| 1  2 |  |  | ۷-a |
|  |  |  | ۷-b |
| ---- | *مجموعه :حداقل ..................حداکثر------------*  *نارینه : حداقل....................حداکثر------------*  *ښځینه : حداقل ...................حداکثر------------* | ستاسی په نظر څو کسان چه نشه یی توکی تزریق کوی د **کندهار** په ښار کی ژوند کوي؟  نوټ: که تعداد ډیرکم یا زیات وه (مثلا ددی ښار د نفوس څخه زیات) په دی صورت کی د شخص څخه وغواړئ بیا فکر وکړي. همدارنګه د نارینه او ښخینه تعدا باید د مجموعی تعداد سره برابر شی. | ۸ |

د همدی هاټ سپاټ په هکله سوالونه

| **کود** | **جواب** | **سوال** | **Q#** |
| --- | --- | --- | --- |
| 1  2  3  4  5  6  7  8 | 1 – 5 ځل  6 – 10 ځل  11 – 15 ځل  16 – 20 ځل  21 – 25 ځل  26+ ځل  نه پوهیږم  د ځواب ورکولو څخه یی انکار وکړ | په تیرو ۳۰ ورځو کی تاسو څو ځلې ددی هاټ سپاټ څخه لیدنه کړیده؟ | ۹ |
|  | ورځ............................................................  وخت............................................................  ټاکلې نیټه: (ورځ/میاشت/کال)................................. | په کومه ورځ په دی هاټ سپاټ کی تر ټولو زیات د نشه یی توکو استعمالونکي اویا د نشه یی توکو تزریق کوونکي راټولیږي | ۱۰ |
|  | نارینه:......................................................  ښځینه:..................................................... | تاسی په تیرو ۷ ورځو کی څو مختلف کسان چه نشه یی توکي تزریق کوي په دی هاټ سپاټ کی ولیدل؟ | ۱۱ |
|  | نارینه:......................................................  ښځینه:..................................................... | تاسی په تیرو ۳۰ ورځو کی څو مختلف کسان چه نشه یی توکي تزریق کوي په دی هاټ سپاټ کی ولیدل؟ | ۱۲ |
| 1  2  3  4  5  6 | مفت سرنج یا ستن  مفت کنډم  د HIV مفت معاینات او مشاوره  مفت تعلیمی او وقایوی معلومات  نور لطفا ذکر یی کړئ ________________)  هیڅ یو، ترڅو چه زه پوهیږم | د تیری میاشتی په دوران کی په دی هاټ سپاټ کی لاندی کوم خدمات تاسی ته ارائه شویدی؟ | ۱۳ |
| 1  2  3 | بلی  نخیر  نه پوهیږم | ایا پولیس په تیره میاشت کی دی هاټ سپاټ ته کوم ځل راغلی وه؟ | ۱۴ |

د د دری نورو هاټ سپاټونو په هکله سوالونه چه دا شخص اکثر د هغوی نه لیدنه کوي

| **Code** | **جواب** | **سوال** | **Q#** |
| --- | --- | --- | --- |
| 1  2  3  4  5 | زه نورو هاټ سپاټونو ته نه ځم (۲۲ سوال ته لاړ شئ)  یو هاټ سپاټ  دوه هاټ سپاټ  دری هاټ سپاټ  د دری هاټ سپاټوڅخه اضافه (څو: ________) | تاسی اکثرا د نشه یی توکو د تزریق او یا هم د هغه ملګرو د لیدلو لپاره چه نشه یی توکی تزریق کوي څو نورو هاټ سپاټونو ته ځی؟ | ۱۵ |
|  | د هاټ سپاټ نوم:....................................  ادرس....................................................... | د هغه هاټ سپاټ نوم او ادرس چه تاسی اکثر د نشه یی توکو د تزریق لپاره او یا هم د هغه ملګرو د لیدلو لپاره چه نشه یی توکی تزریق کوي ورځئ؟ | ۱۶ |
| 1  2  3  4  5  6  7  8  9 | 1 – 5 ځل  6 – 10 ځل  11 – 15 ځل  16 – 20 ځل  21 – 25 ځل  26+ ځل  نه پوهیږم  د ځواب ورکولو څخه یی انکار وکړ  د تطبیق وړ نه دی | په تیرو ۳۰ ورځو کی تاسو څو ځلې ددی هاټ سپاټ څخه لیدنه کړیده؟ | ۱۷ |
|  | د هاټ سپاټ نوم:....................................  ادرس....................................................... | د دوهم هاټ سپاټ نوم او ادرس چه تاسی اکثر د نشه یی توکو د تزریق لپاره او یا هم د هغه ملګرو د لیدلو لپاره چه نشه یی توکی تزریق کوي ورځئ؟ | ۱۸ |
| 1  2  3  4  5  6  7  8  9 | 1 – 5 ځل  6 – 10 ځل  11 – 15 ځل  16 – 20 ځل  21 – 25 ځل  26+ ځل  نه پوهیږم  د ځواب ورکولو څخه یی انکار وکړ  د تطبیق وړ نه دی | په تیرو ۳۰ ورځو کی تاسو څو ځلې ددی هاټ سپاټ څخه لیدنه کړیده؟ | ۱۹ |
|  | د هاټ سپاټ نوم:....................................  ادرس....................................................... | د دریم هاټ سپاټ نوم او ادرس چه تاسی اکثر د نشه یی توکو د تزریق لپاره او یا هم د هغه ملګرو د لیدلو لپاره چه نشه یی توکی تزریق کوي ورځئ؟ | ۲۰ |
| 1  2  3  4  5  6  7  8  9 | 1 – 5 ځل  6 – 10 ځل  11 – 15 ځل  16 – 20 ځل  21 – 25 ځل  26+ ځل  نه پوهیږم  د ځواب ورکولو څخه یی انکار وکړ  د تطبیق وړ نه دی | په تیرو ۳۰ ورځو کی تاسو څو ځلې ددی هاټ سپاټ څخه لیدنه کړیده؟ | ۲۱ |

سلوکی سوالونه

| 1  2  3  4  5  6 | مجرد  متاهل او د خپلی ښځی سره ژوند کوم  متاهل اما د خپلی ښځی سره ژوند نه کوم  واده می نه دی کړی اما د خپل ملګرې سره اوسیږم  طلاق شوی/جدا شوی  کونډ/کونډه | ستاسی د تاهل فعلی وضعیت څه شی دی؟ | ۲۲ |
| --- | --- | --- | --- |
| 1  2 | بلی  نخیر (**۲۷ سوال ته لاړ شئ**) | **(دنارینه لپاره**) آیا تر اوسه پوری تاسی مقعدی یا دخولی د لاری جنسی رابطه د یو بل نارینه سره نیولی ده؟ | ۲۳ |
| 1  2 | بلی  نخیر | **(دنارینه لپاره**) آیا په تیرو ۱۲ میاشتو کی دی مقعدی یا دخولی د لاری جنسی رابطه د یو بل نارینه سره نیولی ده؟ | ۲۴ |
| 1  2  3 | بلی  نخیر, ما د پیسو یا نشه یی توکو په مقابل کی جنسی رابطه دلوده، اما په تیرو ۱۲ میاشتو کی نخیر.  نخیر, ما هیڅکله د پیسو یا نشه یی توکو په مقابل کی جنسی رابطه نه دلوده، | ***(*دنارینه لپاره**)***)*** آیا په تیرو ۱۲ میاشتو کی تاسی مقعدی یا دخولی د لاری جنسی رابطه د پیسو یا نشه یی توکی په مقابل کی تبادله کړیده؟  **که چیری ځواب نخیر وي، ایا نخیر د تل لپاره یا هم یواځی د تیرو ۱۲ میاشتو لپاره؟** | ۲۵ |
| 1  2  3 | بلی  نخیر, ما د پیسو یا نشه یی توکو په مقابل کی جنسی رابطه دلوده، اما په تیرو ۳ میاشتو کی نخیر.  نخیر, ما هیڅکله د پیسو یا نشه یی توکو په مقابل کی جنسی رابطه نه دلوده، | ***(*د ښځو لپاره**)***)*** آیا په تیرو ۳ میاشتو کی تاسی مهبلی، مقعدی یا دخولی د لاری جنسی رابطه د پیسو یا نشه یی توکی په مقابل کی درلوده؟  **که چیری ځواب نخیر وي، ایا نخیر د تل لپاره یا هم یواځی د تیرو ۳ میاشتو لپاره؟** | ۲۶ |
| 1  2 | بلی  نخیر**( د پای برخی ته لاړشئ)** | معاینات کړیدي ؟HIVایا تر اوسه مو د | ۲۷ |
| 1  2  3 | په تیرو ۱۲ میاشتو کی  1-2 کاله مخکی  تر ۲ کالو اضافه تر | مو څه وخت کړیدي؟HIVاخرین معاینات د | ۲۸ |
| 1  2 | بلی  نخیر**( د پای برخی ته لاړشئ)** | په نتیجه مو پوهیږی؟HIVایا د | ۲۹ |
| 1  2  3 | HIV منفی  HIV مثبت  راحت نه احساسوی چه خپله نتیجه بیان کړی | اگر شما راحت هستین میشود بگوید، حالت شما چیست؟ که تاسی راحت یاست، کولای شی چه ووایاست ستاسی د HIV نتیجه څه ده | ۳۰ |

پای برخه

|  | د هاټ سپاټ نمبر |
| --- | --- |
|  | د ګډونکوونکی نمبر |
|  | د مرکه کوونکی نوم |
|  | د فورمی د تمیل نیټه (ورځ/میاشت/کال) |
