# Supplementary material for: Mapping and population size estimates of people who inject drugs in Afghanistan in 2019: Synthesis of multiple methods
Source: PLoS One. 2022 Jan 28;17(1):e0262405. doi: 10.1371/journal.pone.0262405 (PMC8797259; doi:10.1371/journal.pone.0262405)
Supplement: S4 Appendix — (DOCX) [file pone.0262405.s004.docx]

**Appendix 4.** Unduplicated counts of people who inject drugs (PWID) reached by specific programs in each city, Afghanistan.

| **City** | **ADAA** | **BDN** | **SHRO** | **YHDO** | **MoPH DIC** |
| --- | --- | --- | --- | --- | --- |
| Kabul |  |  |  |  | FreeNeedleSyringe=898 |
| Herat |  | FreeCondom=68 |  |  | SyphilisSTItreatment=30  FreeCondom=181  FreePreventionEdu=181  HBSHCVtest=99  FreeNeedleSyringe=181  DIC=181 |
| Mazar |  | FreePreventionEdu=470 |  |  |  |
| Jalalabad | SyphilisSTItreatment=19  HIVtest=236  FreePreventionEdu=236  HBSHCVtest=236  FreeNeedleSyringe=236  Syphilistest=236  DIC=236 |  |  |  |  |
| Kunduz |  |  |  | HIVtest=735  FreeCondom=735  HBSHCVtest=735  FreeNeedleSyringe=735 |  |
| Faizabad |  |  |  | FreePreventionEdu=74 |  |
| Kandahar | FreePreventionEdu=902  DIC=902  Syphilistest=902 |  |  |  |  |
| Zaranj |  |  | FreeNeedleSyringe=684  HBSHCVtest=594 |  |  |

ADA Agency for Assistance and Development for Afghanistan; BDN Bakhter Development Network; SHRO Shahamat Health and Rehabilitation Organization; YHDO Youth Health and Development Organization; MOPH DIC Ministry of Public Health Drop in Center
