# Supplementary material for: Mapping and population size estimates of people who inject drugs in Afghanistan in 2019: Synthesis of multiple methods
Source: PLoS One. 2022 Jan 28;17(1):e0262405. doi: 10.1371/journal.pone.0262405 (PMC8797259; doi:10.1371/journal.pone.0262405)
Supplement: S5 Appendix — (DOCX) [file pone.0262405.s005.docx]

**Appendix 5.** Proxy indicators to extrapolate the population size estimations for people who inject drugs (PWID) from study sites to non-study sites, Afghanistan, 2019.

| **ID** | **Province** | **City/Town** | **Native** | **Administration** | **Included in PSE2019** | **PWID in PSE 2019** | **Total Population 2018** | **Urban Population 2018** | **Adult population 15-64 years** | **Adult Male population 15-64 years** |
| --- | --- | --- | --- | --- | --- | --- | --- | --- | --- | --- |
| 1 | Kunar | Asadābād | اسعد اباد | KNR | 0 | 0 | 36,864 | 15,178 | 8204 | 4,141 |
| 2 | Samangan | Aybak | ایبک | SMG | 0 | 0 | 114,061 | 32,325 | 17490 | 9,147 |
| 3 | Bamyan | Bāmiyān | بامیان | BAM | 0 | 0 | 91,428 | 14,054 | 7604 | 3,977 |
| 4 | Panjsher | Bāzārak | بازارک | PNJ | 0 | 0 | 20,531 | - | - | - |
| 5 | Ghor | Chaghcharān | چغچران | GHW | 0 | 0 | 148,234 | 7,730 | 4181 | 2,149 |
| 6 | Parwan | Chārīkār | چا ریکار | PWN | 0 | 0 | 194,471 | 62,821 | 33980 | 17,584 |
| 7 | Farah | Farāh | فراه | FRH | 0 | 0 | 123,135 | 40,757 | 22032 | 11,150 |
| 8 | Badakhshan | Faizabad | فیض اباد | BDS | 1 | 1 | 74,031 | 36,821 | 19910 | 10,189 |
| 9 | Paktia | Gardīz | گردیز | PTY | 0 | 0 | 92,038 | 26,983 | 14592 | 7,482 |
| 10 | Ghazni | Ghaznī | غزنی | GHZ | 0 | 0 | 179,459 | 65,772 | 35567 | 18,228 |
| 11 | Herat | Herāt | هرات | HRT | 1 | 1 | 538,673 | 538,673 | 291336 | 150,110 |
| 12 | Nangarhar | Jalalabad | جلال اباد | NGH | 1 | 1 | 255,012 | 255,012 | 137868 | 70,099 |
| 13 | Kabul | Kabul | کابل | KBL | 1 | 1 | 4,117,414 | 4,117,414 | 2225687 | 1,125,624 |
| 14 | Kandahar | Kandahar | کندهار | KDH | 1 | 1 | 596,423 | 490,820 | 265355 | 134,936 |
| 15 | Khost | Khost | خوست | KHW | 0 | 0 | 150,599 | 12,789 | 6914 | 3,504 |
| 16 | Kunduz | Kunduz | کندز | KDZ | 1 | 1 | 347,765 | 177,563 | 96000 | 48,871 |
| 17 | Helmand | Lashkar Gah | لشکر گاه | HLM | 0 | 0 | 186,708 | 85,119 | 46091 | 24,733 |
| 18 | Kapisa | Mahmud-i-Raqi | محمودراقی | KAP | 0 | 0 | 70,206 | 1,686 | 908 | 400 |
| 19 | Wardak | Maidan Shar | میدان شهر | WRD | 0 | 0 | 44,180 | 3,375 | 1826 | 955 |
| 20 | Balkh | Mazar-i-Sharif | مزار شریف | BLK | 1 | 1 | 454,457 | 454,457 | 245720 | 125,377 |
| 21 | Laghman | Mehtarlam | مهترلام | LGM | 0 | 0 | 141,635 | 5,622 | 3038 | 1,514 |
| 22 | Faryab | Maymana | میمنه | FYB | 0 | 0 | 91,490 | 91,490 | 49471 | 25,314 |
| 23 | Daikundi | Nīlī | نیلی | DAK | 0 | 0 | 41,367 | - | - | - |
| 24 | Nuristan | Parun | پارون | NUR | 0 | 0 | 14,755 | - | -0 | - |
| 25 | Logar | Pol-e Alam | پل علم | LWG | 0 | 0 | 115,626 | 6,044 | 3268 | 1,672 |
| 26 | Baghlan | Pol-e Khomr | پلخمری | BGL | 0 | 0 | 233,009 | 117,911 | 63754 | 32,560 |
| 27 | Zabul | Qalāt | قلا ت | ZAB | 0 | 0 | 43,230 | 12,403 | 6706 | 3,425 |
| 28 | Badghis | Qal'eh-ye Now | قلعه نو | BDG | 0 | 0 | 72,619 | 16,161 | 8738 | 4,458 |
| 29 | Sar-e-Pul | Sar-e Pol | سرپل | SPL | 0 | 0 | 170,499 | 33,588 | 18159 | 9,236 |
| 30 | Paktika | Sharana | شرنه | PTK | 0 | 0 | 62,495 | 4,816 | 2603 | 1,323 |
| 31 | Jawzjan | Sheberghān | شبر غان | JWJ | 0 | 0 | 184,964 | 89,242 | 48268 | 24,908 |
| 32 | Takhar | Tāloqān | تالقان | TKR | 0 | 0 | 248,830 | 79,967 | 43258 | 22,437 |
| 33 | Urugan | Tarinkot | ترینکوت | ORU | 0 | 0 | 112,283 | 7,449 | 4029 | 2,070 |
| 34 | Nimruz | Zaranj | زرنج | NIM | 1 | 1 | 62,698 | 28,951 | 15656 | 8,039 |

| **ID** | **Province** | **Adult Female population 15-64 years** | **Youth Literacy rate (15-24 years) % in 2017 on provincial basis** | **Literacy Rate of Population age 15 and over % in 2017 on provincial basis** | **Population per square kilometer** | **Percentage of persons in poverty in 2017** | **Percentage of population unemployed in 2017** | **At least one major road exit in or within 10 kilometers of the city border** | **Drug trafficking routes** | **Borders with cities/town that produce and traffic drugs** | **HIV reported cases in 2018 per province** |
| --- | --- | --- | --- | --- | --- | --- | --- | --- | --- | --- | --- |
| 1 | Kunar | 4,063 | 55.8 | 38.2 | 439.862947 | 61.8 | 25.9 | 2 | 2 | 2 | 0 |
| 2 | Samangan | 8,343 | 42.8 | 24 | 53.202252 | 88.2 | 31.6 | 1 | 1 | 2 |  |
| 3 | Bamyan | 3,627 | 64.9 | 40.9 | 50.871528 | 61.3 | 37.4 | 2 | 2 | 2 |  |
| 4 | Panjsher | - | 76.9 | 49.4 | 52.21127 | 25.1 | 45.4 | 2 | 2 | 2 |  |
| 5 | Ghor | 2,032 | 48.6 | 25.2 | 15.652987 | 60.5 | 10.8 | 2 | 1 | 1 |  |
| 6 | Parwan | 16,396 | 62.1 | 41.4 | 753.413939 | 56.3 | 33.3 | 1 | 1 | 2 | 0 |
| 7 | Farah | 10,882 | 37.1 | 24.6 | 33.995793 | 42.6 | 28.1 | 1 | 1 | 1 | 2 |
| 8 | Badakhshan | 9,721 | 46.4 | 28 | 149.028505 | 81.5 | 7.9 | 2 | 1 | 1 | 2 |
| 9 | Paktia | 7,110 | 42.6 | 26 | 135.876888 | 73.7 | 16.2 | 1 | 1 | 2 | 5 |
| 10 | Ghazni | 17,339 | 62.5 | 40.8 | 470.163156 | 42.6 | 20.5 | 1 | 1 | 2 | 1 |
| 11 | Herat | 141,226 | 52.5 | 34.5 | 2241.876783 | 47.9 | 31.3 | 1 | 1 | 1 | 14 |
| 12 | Nangarhar | 67,769 | 51.2 | 33.5 | 14425.999744 | 50.7 | 12.9 | 1 | 1 | 1 | 26 |
| 13 | Kabul | 1,100,063 | 74.4 | 54.2 | 10716.412791 | 34.3 | 25.3 | 1 | 1 | 1 | 77 |
| 14 | Kandahar | 130,419 | 27.1 | 15.8 | 843.601875 | 80.7 | 12.4 | 1 | 1 | 1 | 11 |
| 15 | Khost | 3,410 | 41.5 | 27.7 | 360.408682 | 29.7 | 5.5 | 1 | 1 | 2 | 15 |
| 16 | Kunduz | 47,129 | 47.6 | 29.6 | 583.58736 | 66.4 | 32.8 | 1 | 1 | 2 | 6 |
| 17 | Helmand | 21,358 | 21.4 | 14.8 | 98.871501 | 88.5 | 13.8 | 2 | 1 | 1 |  |
| 18 | Kapisa | 508 | 70.3 | 49.1 | 407.235576 | 45.2 | 23 | 2 | 1 | 1 |  |
| 19 | Wardak | 871 | 55.6 | 37.5 | 210.424908 | 60.4 | 15.9 | 1 | 1 | 2 |  |
| 20 | Balkh | 120,343 | 57.7 | 37.9 | 6422.773608 | 48.8 | 31.1 | 1 | 1 | 1 | 11 |
| 21 | Laghman | 1,524 | 47.5 | 32.5 | 197.869317 | 76.5 | 17.8 | 2 | 1 | 1 |  |
| 22 | Faryab | 24,157 | 33.5 | 20.7 | 992.961619 | 55.8 | 17.3 | 2 | 2 | 1 |  |
| 23 | Daikundi | - | 56.4 | 34.8 | 69.866744 | 90.2 | 46.4 | 2 | 2 | 1 |  |
| 24 | Nuristan | - | 41 | 27.5 | 9.782175 | 60.9 | 13.3 | 2 | 2 | 2 |  |
| 25 | Logar | 1,596 | 38.4 | 26.2 | 102.317321 | 39 | 16.3 | 1 | 1 | 2 |  |
| 26 | Baghlan | 31,194 | 50.3 | 29.9 | 334.859931 | 31.2 | 45.1 | 1 | 1 | 2 | 1 |
| 27 | Zabul | 3,281 | 42.3 | 17.2 | 22.582245 | 81.4 | 21.9 | 1 | 1 | 1 |  |
| 28 | Badghis | 4,280 | 24.8 | 17.4 | 92.66479 | 56.8 | 26.9 | 1 | 1 | 1 |  |
| 29 | Sar-e-Pul | 8,923 | 43.3 | 26.6 | 69.74742 | 56.6 | 39 | 2 | 1 | 1 |  |
| 30 | Paktika | 1,280 | 46.6 | 34.1 | 129.178396 | 12.8 | 24 | 1 | 2 | 2 |  |
| 31 | Jawzjan | 23,360 | 41.9 | 25.6 | 94.705275 | 65.3 | 17.3 | 1 | 1 | 1 |  |
| 32 | Takhar | 20,821 | 52.9 | 68.1 | 298.91749 | 70.2 | 21.7 | 1 | 1 | 2 |  |
| 33 | Urugan | 1,959 | 45.7 | 33.4 | 56.82095 | 69.6 | 11.9 | 2 | 1 | 1 | 1 |
| 34 | Nimruz | 7,617 | 48.7 | 31.2 | 36.161327 | 58.7 | 18.3 | 1 | 1 | 1 | 12 |

| **ID** | **Province** | **Total HIV Cases per province (1989-2018)** | **HCV Reported Cases at the end of 2108** | **Other STI reported cases on provincial level** | **Drugs in tones that seized by police in past year on Provincial Level** | **Number of arrests for drug-related crimes by police in past year** | **Number of needle/syringes distributed in past year** | **Poppy Cultivation in Hectare per province in 2018** | **Opium production in tons per province 2018** |
| --- | --- | --- | --- | --- | --- | --- | --- | --- | --- |
| 1 | Kunar | 54 | 6 | 1 | 0.08524 | 24 | 0 | 1,732 | 43 |
| 2 | Samangan | 7 |  |  | 0.079265 | 15 |  | 238 | 6 |
| 3 | Bamyan | 18 |  |  | 0.000375 | 5 |  | Poppy Free | Poppy Free |
| 4 | Panjsher | 0 |  |  | 0.105506 | 13 |  | Poppy Free | Poppy Free |
| 5 | Ghor | 8 |  |  | 0.137436 | 13 |  | 3,574 | 80 |
| 6 | Parwan | 14 | 22 | 1 | 2.866271 | 88 | 1712 | Poppy Free | Poppy Free |
| 7 | Farah | 23 | 27 | 35 | 22.39307 | 49 | 275584 | 10,916 | 243 |
| 8 | Badakhshan | 72 | 15 | 3 | 1.971207 | 65 | 48189 | 7,703 | 262 |
| 9 | Paktia | 34 | 15 | 3 | 75.80789 | 48 | 26007 | Poppy Free | Poppy Free |
| 10 | Ghazni | 40 | 114 | 0 | 0.042424 | 45 | 365995 | 373 | 9 |
| 11 | Herat | 346 | 28 | 9 | 0.882646 | 416 | 63716 | 595 | 13 |
| 12 | Nangarhar | 400 | 14 | 8 | 84.69316 | 372 | 147412 | 17,177 | 428 |
| 13 | Kabul | 1036 | 86 | 10 | 15.50805 | 1599 | 219199 | 484 | 11 |
| 14 | Kandahar | 128 | 37 | 8 | 0.729437 | 197 | 115035 | 23,410 | 564 |
| 15 | Khost | 75 | 87 | 20 | 0.196766 | 112 | 0 | Poppy Free | Poppy Free |
| 16 | Kunduz | 53 | 198 | 5 | 1.11313 | 62 | 345472 | Poppy Free | Poppy Free |
| 17 | Helmand | 15 |  |  | 4.994102 | 133 |  | 136,798 | 3,297 |
| 18 | Kapisa | 1 |  |  | 0.016165 | 44 |  | 386 | 10 |
| 19 | Wardak | 16 |  |  | 1.240815 | 11 |  | Poppy Free | Poppy Free |
| 20 | Balkh | 164 | 17 | 46 | 0.243876 | 154 | 306170 | 8,532 | 225 |
| 21 | Laghman | 16 |  |  | 0.28449 | 50 |  | 2,092 | 52 |
| 22 | Faryab | 16 |  |  | 0.113239 | 34 |  | 8,175 | 216 |
| 23 | Daikundi | 13 |  |  | 0.02365 | 41 |  | 747 | 18 |
| 24 | Nuristan | 0 |  |  | 0.00006 | 1 |  | Poppy Free | Poppy Free |
| 25 | Logar | 10 |  |  | 27.0954 | 15 |  | Poppy Free | Poppy Free |
| 26 | Baghlan | 27 |  |  | 15.34223 | 111 |  | 1,076 | 28 |
| 27 | Zabul | 17 |  |  | 0.062669 | 12 |  | 2,581 | 62 |
| 28 | Badghis | 1 |  |  | 0.019402 | 42 |  | 6,973 | 156 |
| 29 | Sar-e-Pul | 23 |  |  | 1.689378 | 28 |  | 660 | 17 |
| 30 | Paktika | 32 |  |  | 31.0964 | 22 |  | Poppy Free | Poppy Free |
| 31 | Jawzjan | 7 |  |  | 1.602728 | 43 |  | 338 | 9 |
| 32 | Takhar | 36 |  |  | 0.256318 | 87 |  | 251 | 9 |
| 33 | Urugan | 3 |  |  | 0.06418 | 4 |  | 18,662 | 450 |
| 34 | Nimruz | 28 | 38 | 9 | 16.17137 | 120 | 202637 | 9,115 | 203 |

Based on Lasso regression and data overlapping, we selected the “Percentage of population unemployed in 2017” as the only proxy indicator for the extrapolation. The following Poisson models were used for prediction formula was used to extrapolate the point, upper and lower bound estimate of the male PWID population size from the eight study cities to other unobserved cities:

- Point estimate of the population size for male PWID = exp( -4.325 - 0.0269 x Proportion Unemployed) x Adult Male Population
- Lower limit estimate of the population size for male PWID = exp( -4.584 - 0.029 x Proportion Unemployed) x Adult Male Population
- Upper limit estimate of the population size for male PWID = exp( -4.109 - 0.024 x Proportion Unemployed) x Adult Male Population

Data sources for predicators listed in the above table:

- Annual Opium Poppy Survey 2018- UNODC <https://www.unodc.org/documents/crop-monitoring/Afghanistan/Afghanistan_opium_survey_2018.pdf> Afghanistan Statistical Yearbook 2018-19 – NSIA
- <https://www.nsia.gov.af:8080/wp-content/uploads/2019/11/Afghanistan-Statistical-Yearbook-2018-19_compressed.pdf>
- Drug seizures and detainee’s data, Counter Narcotic Police of Afghanistan (CNPA)
- All other data provided by the National program for HIV, Hepatitis B and C and Syphilis
